# Causal association among smoking, bitter beverage consumption, and risk of osteoporosis: a two-sample mendelian randomization-based study

**DOI:** 10.1186/s41065-025-00371-1

**Published:** 2025-01-24

**Authors:** Yanqian Wu, Jianqian Chao, Min Bao, Na Zhang, Leixia Wang

**Affiliations:** 1 Health Management Research Center, School of Public Health, Southeast University, 87 Dingjiaqiao Road, Gulou District, Nanjing, 210009 P.R. China; 2 Department of Nursing Research Institute, Affiliated Hospital of Medical School, Nanjing Drum Tower Hospital, Nanjing University, Nanjing, 210008 P.R. China

**Keywords:** Osteoporosis, Fracture, Smoking, Mendelian randomization, Bitter beverage consumption

## Abstract

**Objectives:**

Two-sample MR methods were employed to analyze the impact of smoking and bitter beverage consumption on the risk of osteoporosis and osteoporosis with pathological fractures, in order to assess the causal association.

**Methods:**

Publicly available genome-wide association study summary data were analyzed using MR methods. The exposures investigated were smoking (smoking per day, smoking initiation, and lifetime smoking index) and bitter beverages (coffee, tea, bitter alcoholic beverages, bitter non-alcoholic beverages, and total bitter beverages). The outcomes examined were the risk of osteoporosis and osteoporosis with pathological fractures. The inverse-variance weighted (IVW) method was used as the main statistical model. The stability and reliability of the results were verified by the Cochran’s Q test, the Egger-intercept test, and the leave-one-out analysis.

**Results:**

Smoking per day was causally associated with the risk of osteoporosis OR = 1.417, 95% CI = 1.119–1.794, *P* = 0.003), and lifetime smoking index had a possible genetic causal association with the risk of osteoporosis with pathological fractures (OR = 4.187, 95% CI = 1.909–9.184, *P* < 0.001). No genetic causal association was found between smoking initiation or lifetime smoking index and the risk of osteoporosis (*P* > 0.05). No genetic causal association was identified between smoking per day or smoking initiation and the risk of osteoporosis with pathological fractures (*P* > 0.05). Total and bitter non-alcoholic beverage consumption showed a potential effect on the risk of osteoporosis (OR = 3.687, 95% CI = 1.535–8.858, *P* = 0.003 and OR = 3.040, 95% CI = 1.466–6.304, *P* = 0.002, respectively).

**Conclusions:**

This study found smoking raises the risk of osteoporosis and osteoporosis with pathological fractures based on genetics. Certain bitter beverages are linked to an increased osteoporosis risk.

**Supplementary Information:**

The online version contains supplementary material available at 10.1186/s41065-025-00371-1.

## Background

Osteoporosis is a prevalent and insidious chronic disease that primarily affects middle-aged and senior-aged individuals [1]. In severe cases, osteoporosis may result in fractures that commonly occur in the wrists, spine, and hips [2]. Its complications, such as pain and bone deformities, can significantly reduce the quality of life and lifespan of older patients [3]. The resulting disabilities in middle-aged and older individuals due to spinal and hip fractures not only reduce their quality of life and lifespan, but also impose significant medical expenses and caregiving burdens [4]. Nonetheless, there are certain factors that can be controlled to reduce the risk of developing osteoporosis, such as alcohol consumption, coffee intake, and smoking [5]. By addressing these controllable factors, individuals can take proactive steps to lower their risk of osteoporosis.

Smoking is a well-known risk factor for various health-related problems, such as lung cancer, respiratory diseases, and cardiovascular disease [6]. Its influence on bone health is also concerning as evidence suggested that smoking causes reduced bone density, which is a significant risk factor for fractures [7]. Observational studies have reported the association of smoking with the increased risk of osteoporosis [8, 9]. To investigate whether smoking history could affect osteoporosis and osteoporotic fractures, Jaramillo et al. studied 3,321 patients, and it was demonstrated that male smokers, whether they have chronic obstructive pulmonary disease (COPD) or not, are at a great risk of low volumetric bone mineral density (vBMD) and vertebral fractures [9]. However, the correlation needs further exploration and confirmation through more extensive population-based studies. Mendelian randomization (MR) is a method that leverages genome-wide association study (GWAS) data to estimate causal effects between risk factors and outcomes [10]. Using genetic variation as an instrumental variable (IV), MR can make causal inferences without relying on randomized controlled trials (RCTs). The approach is well-suited to utilize GWAS data and single-nucleotide polymorphisms (SNPs) to infer the causal effect of exposure on outcomes. The use of genetic markers as IVs reduces the risk of confounding and reverse causation, enabling more robust estimates of causal effects. MR provides a valuable tool for exploring causal relationships in non-experimental data, and its advantages include objectivity, ease of implementation, and the absence of ethical concerns associated with conducting RCTs [10, 11]. MR-based studies have been implemented to establish a causal association between smoking and BMD [12, 13]. In specific demographic groups, a correlation between smoking and the risk of osteoporosis has been identified, while further research is necessary to verify these findings.

There is conflicting evidence regarding the association between consumption of bitter beverages, such as coffee and drink, and the risk of osteoporosis. Several studies have suggested that caffeine could promote bone loss and decrease BMD [14, 15], while others have found no association between coffee, tea, or soft drink consumption and calcium loss as long as calcium intake is normal [16, 17]. Additionally, several studies have shown that moderate tea consumption or wine consumption has a positive effect on bone health [10, 18]. However, due to possible residual confounding and reverse causality, and the lack of high-quality RCTs, it is uncertain whether these observational results are causal. Moreover, studies have mainly concentrated on the association between these beverages and BMD and fractures, with limited investigations on the cumulative effects of bitter alcoholic and non-alcoholic beverages on overall bone health. Therefore, further study is essential to elucidate the potential effects of these bitter beverages on bone health.

In the present study, a two-sample MR analysis was performed to evaluate the possible causal associations of smoking and bitter beverage consumption with the risk of osteoporosis.

## Methods

### Study design

**Fig. 1:**
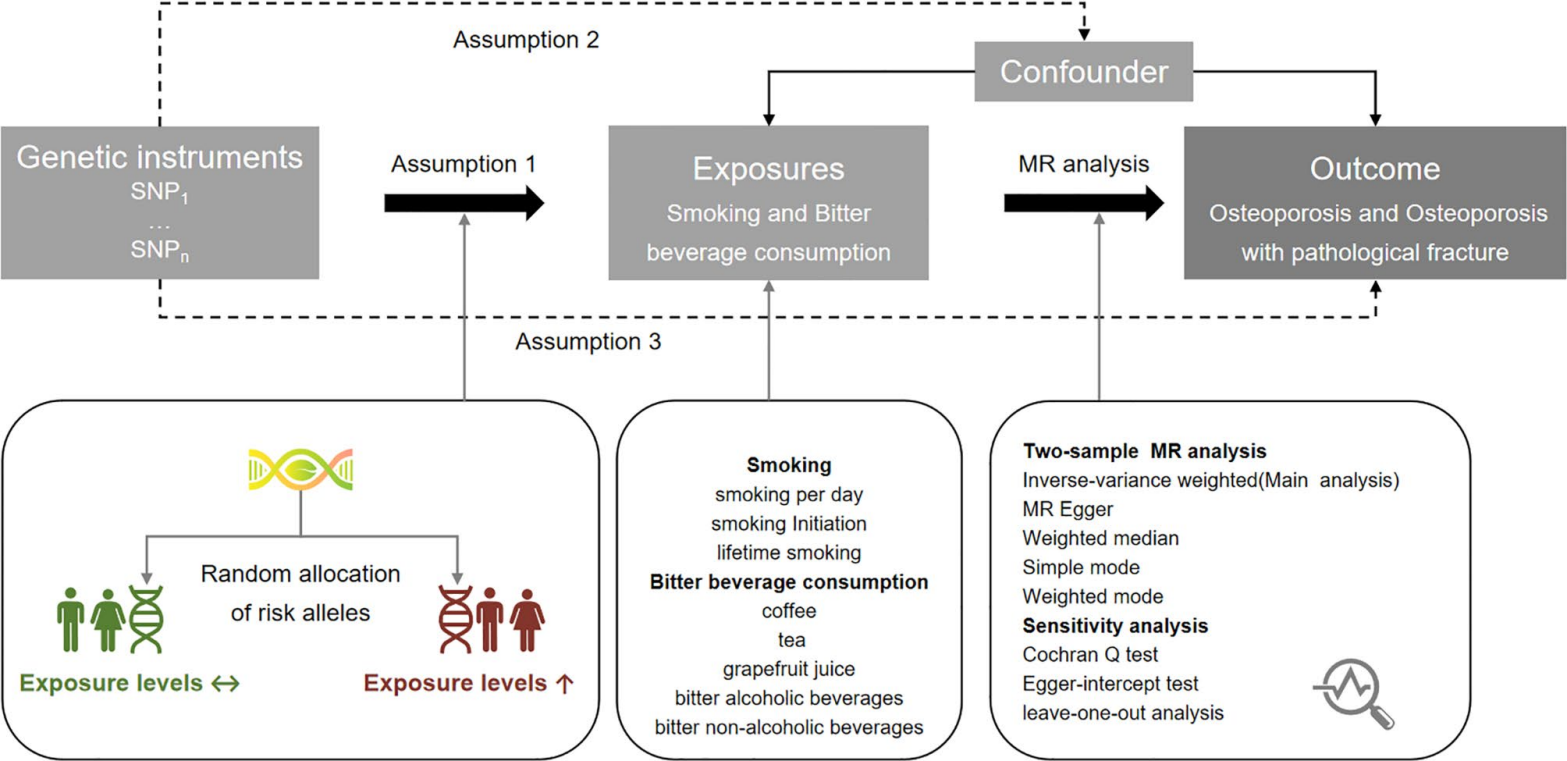
The study design overview

This MR study was performed using summary-level data from published GWASs and the FinnGen consortium. Five algorithms were utilized to carry out MR analysis, including MR Egger, weighted median, inverse-variance weighted (IVW), simple mode, and weighted mode. The assessment of a genetic causal association between the exposure and outcome was based on the results of the IVW analysis, where a *P*-value of less than 0.05 was indicative of a genetic causal association. Finally, the stability and reliability of the results were verified through the Cochran Q test, Egger-intercept test, and leave-one-out analysis. The study design was divided into five parts as follows: (1) identification of genetic variants to serve as IVs for bitter beverage consumption and smoking; (2) acquisition of instrumental SNP outcome summary data from the FinnGen consortium of osteoporosis and osteoporosis with pathological fracture; (3) harmonization of SNP exposure and SNP outcome datasets; (4) conducting two-sample MR analysis; and (5) assessing assumptions of MR analysis, performing sensitivity analysis, and visualizing the results. Figure 1 depicts the study design. The STROBE-MR guideline was used to guide the design of this MR study [19], with the checklist available in the Supplementary Table 1.

### Data source

The exposure variable data for the genetic variants associated with smoking behaviors (smoking per day, smoking initiation, and lifetime smoking index) and bitter beverage consumption (coffee, tea, grapefruit juice, bitter alcoholic beverages, bitter non-alcoholic beverages, and total bitter beverages) were derived from GWASs with enrollment of European participants at the genome-wide significance threshold (*P* < 5 × 10^− 8^) [20, 21]. For smoking behaviors, the SNPs of smoking per day were selected from the Sequencing Consortium of Alcohol and Nicotine Use, along with 23andMe and the UK Biobank. The number of cigarettes smoked per day was taken as the average number of cigarettes smoked per day between current smokers and former smokers, smoking per day included 216,590 European-descent individuals [20]. Smoking initiation was measured as a binary variable, which was coded as “1” if there never was a regular smoker in the life and “2” if there ever was a regular smoker in the life (current or former), and smoking initiation included 1232,091 European-descent individuals [20]. The lifetime smoking index included information related to the duration of smoking, heaviness, and cessation, which were combined into a simulated half-life (τ) constant and a lifetime smoking index, and lifetime smoking index included 462,690 European-descent individuals [20].

Total bitter beverages included coffee, tea, grapefruit juice, and bitter tasting alcoholic beverages (beer/cider, red wine, and liquor) [21]. In the present study, bitter beverages were categorized into alcoholic and non-alcoholic categories, in which coffee and tea were both non-alcoholic beverages. Due to the screening of SNP corresponding *P* values, grapefruit juice data were fully excluded. Finally, five sets of instruments were employed for validation. Bitter beverage consumption information was collected using a 24 h recall questionnaire (OxfordWebQ) in four independent populations of European ancestry: UK Biobank participants, Nurses’Health Study, Health Professionals Follow-up Study, and Women’s Genome Health Study [21]. The SNPs for bitter beverage consumption were obtained from a joint meta-analysis of these studies, in which coffee consumption included 376,923 participants of European ancestry, tea consumption included 376,822 participants of European ancestry, bitter alcoholic beverages included 376,372 participants of European ancestry, bitter non-alcoholic beverages included 122,435 participants of European ancestry, and total bitter beverages included 125,776 participants of European descent.

The outcome variable data on osteoporosis in European populations (including 6,303 cases and 325,717 controls for osteoporosis, and 1,433 cases and 261,098 controls for osteoporosis with pathological fracture) were obtained from the FinnGen consortium. All subtypes of osteoporosis were defined by the code M13 presented in the tenth revision of the International Classification of Diseases (ICD-10). More detailed information on participants, genotyping, attribution, and quality control can be found on the FinnGen website (https://www.finngen.fi/en). Detailed information on data sources, definition, unit, participants included in the analysis, adjusted covariates, and identified SNPs are displayed in Supplementary Table 2.

### Selection of IVs

A series of steps for selecting eligible genetic variants associated with metabolites were performed to control the SNP quality. First, genomic SNPs associated with exposure (*P* < 5 × 10^− 8^) in European-descent individuals were extracted. None of the instrumental SNPs were in linkage disequilibrium (LD). Next, we aggregated SNPs in LD by performing the clumping process (R^2^ ≥ 0.001 and within 10 mb). Meanwhile, the missing SNPs in the LD control group were also excluded. SNPs with a minor allele frequency (MAF) < 0.01 were deleted, and SNPs with an F statistic less than 10 were removed. Finally, some SNPs were used as IVs for smoking and bitter beverage consumption, and the detailed information is presented in Supplementary Table 3.

### Statistical analysis

The IVW method with a fixed-effects model was used as the main statistical model. Four other methods, including the MR Egger, weighted median, simple mode, and weighted mode were utilized, and *P* < 0.05 was considered as a suggestive association. A series of sensitivity analyses, including Cochran’s Q test and MR-Egger intercept test were conducted. The leave-one-out analysis was carried out to determine whether a SNP could exert undue influence on the results of the MR analysis. SNPs with a MAF < 0.01 were deleted, SNPs with an F statistic less than 10 were removed, R^2^ was calculated as follows: R^2^ = 2×EAF*(1 − EAF)*Beta^2^ and the F-statistic for each SNP was calculated as follows: F=(*N* − 2)*R^2^/(1 − R^2^) [20]. The MR analysis was considered to be free of heterogeneity/pleiotropy when *P* > 0.05. In such cases, the reliability of the results of IVW analysis was confirmed. To assess the risk of osteoporosis and osteoporosis with pathological fractures, odds ratios (ORs) and 95% confidence intervals (CIs) were calculated. The analysis was performed using R 4.2.1 software (R Development Core Team, Vienna, http://www.R-project.org). The statistical analysis was carried out using the “TwoSampleMR” package.

### Sensitivity analysis

For the identified significant estimates (IVW *P* < 0.05), sensitivity analysis was conducted to evaluate any bias of the MR assumptions. For this purpose, the Cochran Q test, the Egger-intercept test, and the leave-one-out analysis were used. The heterogeneity test mainly examines the differences between IVs. If IVs significantly differ from each other, they may be highly heterogeneous. The Cochran Q test was employed to detect the existence of heterogeneity. The pleiotropy test primarily examines whether there are multiple IVs exhibiting horizontal pleiotropy, as indicated by the intercept term in the MR Egger method. If the intercept term is significantly different from 0, it indicates the existence of horizontal pleiotropy. The leave-one-out sensitivity test is primarily used to compute the MR results for the remaining IVs after systematically removing one IV at a time. If the MR results estimated by other IVs and the total results are significantly different after removing a certain IV, it indicates that MR results are sensitive to the IV.

## Results

A total of 485 SNPs were included in this study after removing the IVs with linkage imbalance (Supplementary Table 3). The number of SNPs for each variable ranged from 3 to 301. F statistics for SNPs were all over 10, indicating that there was low evidence of weak instrumental bias in this study.

### The causal association between smoking and the risk of osteoporosis

Using the IVW and weighted median methods, it was found that there was a causal association between smoking per day and the risk of osteoporosis (OR = 1.417, 95% CI = 1.119–1.794, *P =* 0.003). Weighted median method (OR = 1.393, 95%CI = 1.010–1.923, *P* = 0.043) and MR-Egger regression (OR = 1.198, 95% CI = 0.678–2.116, *P* = 0.541) presented consistent direction and magnitude, supporting the robustness of the causality (Table 1). The effect of the SNP on daily smoking and the risk of osteoporosis was consistently observed to have the same direction of total effect values across various methods (Supplementary Fig. 1). *P* values for Cochrane’s Q test (the *Q*-*P* values of the IVW and MR–Egger all>0.05) indicated that no heterogeneity was detected (Table 2). There was no significant difference between Egger _ intercept and 0 of MR-Egger (*P* = 0.534), thus, it was suggested that SNPs did not have horizontal pleiotropy (Table 2). The leave-one-out analysis showed that the causal association between smoking per day and the risk of osteoporosis was not driven by any SNPs (Supplementary Fig. 5).

**Table 1:** MR estimates from different methods for assessing the causal effect of osteoporosis

Exposure	Inverse variance weighted method			Weighted median method			MR-Egger regression			Simple mode			Weighted mode		
	OR	95% Cl	*P*	OR	95% Cl	*P*	OR	95% Cl	*P*	OR	95% Cl	*P*	OR	95% Cl	*P*
Smoking															
smoking per day	1.417	1.119–1.794	0.003*	1.393	1.010–1.923	0.043	1.198	0.678–2.116	0.541	1.327	0.740–2.380	0.355	1.400	0.975–2.012	0.086
smoking initiation	1.235	0.987–1.545	0.063	1.286	0.919–1.799	0.141	0.462	0.202–1.056	0.068	0.991	0.336–2.922	0.987	1.089	0.404–2.934	0.866
lifetime smoking index	1.091	0.748–1.591	0.649	1.094	0.645–1.856	0.736	0.938	0.199–4.416	0.936	1.185	0.234–5.991	0.836	1.333	0.298–5.953	0.706
Bitter beverages															
coffee	1.344	0.784–2.305	0.281	1.991	0.955–4.149	0.065	2.171	0.784–6.006	0.145	0.676	0.129–3.516	0.645	2.313	1.220–4.383	0.015
tea	1.723	0.441–6.733	0.433	4.453	1.256–15.785	0.020	10.651	0.225-503.927	0.254	3.810	0.494–29.365	0.223	5.496	1.381–21.860	0.0324
bitter alcoholic beverages	2.431	0.343–17.212	0.373	1.010	0.137–7.419	0.991	261.884	6.525-10509.599	0.031	0.788	0.062–9.949	0.860	0.788	0.089–6.958	0.837
bitter non-alcoholic beverages	3.040	1.466–6.304	0.002*	3.423	1.487–7.883	0.003	3.804	0.386–37.466	0.370	2.383	0.750–7.571	0.237	3.586	1.433–8.975	0.072
Total bitter beverages	3.687	1.535–8.858	0.003*	4.185	1.597–10.966	0.003	4.073	0.227–73.036	0.515	4.325	1.294–14.457	0.140	4.476	1.413–14.172	0.125

**Table 2:** The results of Cochrane’s Q and pleiotropy tests

Exposure	Outcome	Inverse variance weighted method					MR-Egger regression method					Intercept	*P* for intecept
		Beta	SE	*P*	Cochrane’s Q	*P* for Cochrane’s Q	Beta	SE	*P*	Cochrane’s Q	*P* for Cochrane’s Q		
Smoking													
smoking per day	Osteoporosis	0.348	0.120	0.003*	12.637	0.699	0.181	0.290	0.541	12.234	0.661	0.009	0.534
	Osteoporosis with pathological fracture	0.251	0.250	0.316	5.187	0.510	-0.393	-0.603	0.524	13.811	0.539	0.035	0.259
smoking initiation	Osteoporosis	0.211	0.114	0.063	291.395	0.628	-0.771	0.422	0.068	285.536	0.702	0.010	0.016
	Osteoporosis with pathological fracture	0.459	0.238	0.053	295.979	0.554	-0.206	0.879	-0.814	295.359	0.548	0.007	0.431
lifetime smoking index	Osteoporosis	0.087	0.192	0.649	93.215	0.826	-0.063	0.790	0.936	93.176	0.808	0.001	0.844
	Osteoporosis with pathological fracture	1.432	0.400	0.000*	87.543	0.915	1.213	1.643	0.461	87.524	0.903	0.002	0.891
Bitter beverages													
coffee	Osteoporosis	0.296	0.274	0.281	41.463	0.099	0.775	0.519	0.145	39.892	0.106	-0.009	0.285
	Osteoporosis with pathological fracture	-0.051	0.495	0.917	23.798	0.818	-0.849	0.936	0.371	22.791	0.823	0.015	0.323
tea	Osteoporosis	0.544	0.695	0.433	30.601	0.002	2.365	1.967	0.254	28.100	0.003	-0.025	0.343
	Osteoporosis with pathological fracture	-0.843	0.905	0.351	9.189	0.686	-1.052	2.561	0.689	9.182	0.605	0.002	0.932
bitter alcoholic beverages	Osteoporosis	0.888	0.998	0.373	10.469	0.106	5.567	1.883	0.031	3.114	0.682	-0.059	0.042
	Osteoporosis with pathological fracture	0.916	1.539	0.551	5.089	0.532	4.977	3.538	0.218	3.464	0.628	-0.053	0.258
bitter non-alcoholic beverages	Osteoporosis	1.112	0.372	0.002*	0.342	0.951	1.336	1.166	0.370	0.301	0.860	-0.006	0.858
	Osteoporosis with pathological fracture	-0.601	0.784	0.443	3.080	0.379	2.794	2.427	0.368	0.901	0.637	-0.098	0.277
total bitter beverages	Osteoporosis	1.305	0.447	0.003*	0.297	0.861	1.404	1.472	0.515	0.292	0.588	-0.0026	0.954
	Osteoporosis with pathological fracture	-0.306	0.930	0.742	0.693	0.7068	1.946	3.063	0.639	0.098	0.754	-0.060	0.581

However, there was no potential genetic causal association between smoking initiation and the risk of osteoporosis (*P* > 0.05 in all models) (Table 1). In addition, the results of the IVW and other analysis methods did not indicate a causal association between lifetime smoking index and the risk of osteoporosis (*P* > 0.05 in all models) (Table 1).

### The causal association between bitter beverage consumption and the risk of osteoporosis

There was a causal association between total bitter beverage consumption (IVW OR = 3.687, 95% CI = 1.535–8.858, *P* = 0.003; WM OR = 4.185,95% CI = 1.597–10.966, *P* = 0.003) and bitter non-alcoholic beverage consumption (IVW OR = 3.040, 95% CI = 1.466–6.304, *P* = 0.002; WM OR = 3.423, 95% CI = 1.487–7.883, *P* = 0.003) and the risk of osteoporosis (Table 1). The consistency in the direction of the scatter plots of the 3MR model for non-alcoholic bitter beverage and the 3MR model for total bitter beverage with a causal association with the risk of osteoporosis indicated the stability of the research results. The results from other MR methods showed a consistency, while with a nonsignificant direction (Supplementary Figs. 2–3). Moreover, no horizontal pleiotropy was found in the Cochrane’s Q and pleiotropy tests (all intercept *P* values > 0.05), and the heterogeneity analysis found no heterogeneity in the analysis, with *Q*-*P* values > 0.05 in all the analyses (Table 2). The results of the leave-one-out method for total bitter beverages and bitter non-alcoholic beverages showed that no abnormal IV affected the overall results in the two analyses (Supplementary Figs. 6–7). However, no evidence of causal association between coffee or tea consumption and the risk of osteoporosis using the IVW and other methods was found (*P* > 0.05 in all models).

### The causal association between smoking and the risk of osteoporosis with pathological fractures

The IVW and weighted median methods showed a potential causal association between the risk of osteoporosis with pathological fractures and lifetime smoking index (IVW OR = 4.187, 95% CI = 1.909–9.184, *P* < 0.001; WM OR = 4.449, 95% CI = 1.417–1.396, *P* = 0.010). The results of the risk of osteoporosis with pathological fractures are shown in Table 3. The scatter plot of the 3MR model for the correlation between lifetime smoking index and the risk of osteoporosis with pathological fractures showed consistency in the direction of the overall effect value, indicating stable results (Supplementary Fig. 4). In addition, no horizontal multiplicity was found in this analysis (intercept *P =* 0.891). The heterogeneity analysis indicated no heterogeneity in the MR analysis of osteoporosis with pathological fractures and lifetime smoking index (*Q*-*P* value > 0.05 in the IVW and MR-Egger analyses) (Table 2). The leave-one-out analysis showed that the causal relationship of the risk of osteoporosis with pathological fractures and lifetime smoking index was not driven by any SNPs (Supplementary Fig. 8).

**Table 3:** MR estimates from different methods for assessing the causal effect of osteoporosis with pathological fractures

Exposure	Inverse variance weighted method			Weighted median method			MR-Egger regression			Simple mode			Weighted mode		
	OR	95% Cl	*P*	OR	95% Cl	*P*	OR	95% Cl	*P*	OR	95% Cl	*P*	OR	95% Cl	*P*
Smoking															
smoking per day	1.285	0.786–2.101	0.316	1.478	0.747–2.926	0.261	0.674	0.206–2.204	0.524	1.781	0.611-5.187	0.305	1.418	0.681–2.954	0.363
smoking initiation	1.583	0.993–2.525	0.053	1.242	0.608–2.534	0.550	0.813	0.145–4.557	0.814	1.120	0.126–9.932	2.525	0.898	0.117–6.870	0.918
lifetime smoking index	4.187	1.909–9.184	0.000*	4.449	1.417–1.396	0.0105	3.364	0.134–8.425	0.461	3.860	0.206–7.219	0.367	4.088	0.333–5.013	0.273
Bitter beverages															
coffee	0.949	0.359–2.506	0.917	0.862	0.862–3.164	0.823	0.427	0.068–2.682	0.371	0.877	0.078–9.873	0.916	0.796	0.213–2.973	0.737
tea	0.430	0.072–2.539	0.351	0.374	0.034–4.018	0.417	0.349	0.002–5.288	0.689	0.313	0.005–1.651	0.577	0.751	0.043–1.292	0.847
bitter alcoholic beverages	2.499	0.122–5.113	0.551	1.104	0.020–5.926	0.960	145.067	0.141–1.492	0.218	0.126	0.001–4.977	0.522	0.224	0.001–5.925	0.618
bitter non-alcoholic beverages	0.547	0.117–2.549	0.443	0.745	0.125–4.441	0.747	16.362	0.140–1.907	0.368	0.552	0.051–5.914	0.657	0.853	0.149–4.880	0.869
Total bitter beverages	0.736	0.118–4.559	0.742	0.796	0.112–5.654	0.820	7.005	0.017–2.837	0.639	0.601	0.047–7.662	0.733	0.920	0.089–9.477	0.951

However, there was no potential genetic causal association between smoking per day and the risk of osteoporosis with pathological fractures (*P* > 0.05 in all models) (Table 3). Meanwhile, the IVW and other methods showed no potential genetic causal association between the smoking initiation and the risk of osteoporosis with pathological fractures (*P* > 0.05 in all models) (Table 3).

### The causal association between bitter beverage consumption and the risk of osteoporosis with pathological fractures

The results of five different methods found no evidence of a genetic causal association between bitter beverage consumption (coffee, tea, bitter alcoholic beverages, bitter non-alcoholic beverages, total bitter beverages) and the risk of osteoporosis with pathological fractures (*P* > 0.05 in all models), as shown in Table 3.

## Discussion

This study used GWAS data on osteoporosis from 332,020 individuals and data from 262,531 participants with osteoporosis and pathological fractures from the Finnish database. MR analyses revealed the association of genetically predicted increased smoking per day, bitter non-alcoholic beverage consumption, and total bitter beverage consumption with the higher risk of osteoporosis, rather than with coffee consumption or tea consumption. The results also indicated a causal effect of lifetime smoking index on the increased risk of osteoporosis with pathological fractures. However, there was no causal association between bitter beverage consumption and the risk of osteoporosis with pathological fractures.

Smoking has been consistently reported to be associated with the increased risk of osteoporosis in observational studies [22, 23]. Those studies have shown a robust correlation between smoking and BMD in the elderly [8, 9]. It has been revealed that tobacco comprises numerous compounds that pose a threat to smokers’ health [24]. Vaajala et al. conducted a nationwide population-based cohort study in Finland and found that female smokers were more likely to experience bone mass loss and had a greater risk of suffering from fractures compared to non-smokers [8]. The earliest MR on smoking and BMD utilized data from the UK Biobank to investigate the causal association between daily smoking and initial BMD. A significant negative correlation was found between smoking initiation and heel BMD [10]. A recent MR-based study conducted by Yuan et al. indicated a causal association between smoking and the risk of osteoporosis. The study utilized 426,824 bone fractures and estimated BMD from the UK Biobank, in which smoking initiation increased the risk of bone fractures [13]. Larsson et al. conducted a systematic review of MR analyses investigating the connection between smoking and various diseases using initiation or lifetime smoking as instrumental variables. The results indicated that a genetic predisposition to initiation was associated with a higher risk of fracture [25]. These findings emphasized the importance of tobacco cessation for the prevention of osteoporotic fractures and bone density loss. However, there are significant differences between the results of previous studies and the findings of the present study. Firstly, the present study concentrated specifically on patients who had osteoporosis resulting in pathological fractures, in contrast to only assessing any type of fractures. Additionally, the outcome variable was the physician-confirmed diagnosis of osteoporosis in the present study, rather than relying solely on bone density measurements, distinguishing it from previous research. This is important as it confirms the presence of osteoporosis by a medical professional. Moreover, the present study included a more detailed classification of smoking behavior and explored the causal association between smoking per day, initiation of smoking, and lifetime smoking index in association with the risk of osteoporosis and osteoporosis with pathological fractures. This level of specificity enabled us to produce a more comprehensive analysis of smoking’s impact on the condition. In contrast, previous studies lacked this level of precision, potentially leading to less conclusive or complete findings. Overall, the results of the present study may provide new insights into osteoporosis research.

A deeper understanding of the mechanisms underlying smoking’s impact on osteoporosis is crucial. Smoking can negatively affect bone health through several biological pathways. Nicotine, one of the primary components of cigarette smoke, has been shown to impair osteoblast function, which decreases bone formation. At the same time, it increases osteoclast activity, promoting bone resorption [26]. Furthermore, smoking also decreases calcium absorption from the gastrointestinal tract, which further reduces bone mineral density and contributes to increased fracture risk [27]. These findings are consistent with existing literature that demonstrates the negative impact of smoking on bone health, particularly in the elderly population [28, 29]. Our study contributes to this growing body of evidence by focusing specifically on pathological fractures associated with osteoporosis, a clinically significant outcome.

While MR methods are generally robust to confounding, some factors like diet, physical activity, and drug use were not fully considered in this study. These factors might also affect osteoporosis risk. These factors may interact with smoking and other behaviors, influencing bone health. For instance, diets rich in calcium and vitamin D are essential for maintaining bone health, while lack of physical activity can contribute to decreased bone density. Future studies should aim to explore the complex relationships between these lifestyle factors and osteoporosis risk. Such studies could explore how combined lifestyle factors, including diet, exercise, and smoking, interact to influence bone metabolism and osteoporosis risk. This would offer a more comprehensive understanding of the disease and potentially reveal new preventative strategies.

This study utilized GWAS data from European populations, which may limit the generalizability of the results to other populations. While European populations represent a large cohort, it is crucial to consider that genetic and environmental factors can vary across different ethnic groups and regions. Future studies should aim to include more ethnically and geographically diverse populations, such as Asian, African, and Hispanic cohorts, to verify the consistency and robustness of these findings across different demographic and genetic backgrounds. This would help determine whether the observed causal associations are applicable to a broader global population and could provide a more comprehensive understanding of the factors influencing osteoporosis risk.

This study revealed a causal effect of higher bitter non-alcoholic beverage consumption on the increased risk of osteoporosis. The consumption of non-alcoholic beverages in this study mainly refers to the combined consumption of coffee and tea. Studies have pointed out that coffee intake is closely related to postmenopausal osteoporosis, especially in women who consume more than 4 cups/d caffeine after menopause, leading to a significant increase in the risk of bone loss, especially in the lumbar spine, and caffeine may result in an increase in the body’s output of chloride, sodium, magnesium, calcium, and other substances The present MR-based study found no evidence of a causal association between coffee consumption and the risk of osteoporosis, as such, it can be suggested that moderate coffee intake does not appear to be a major contributing factor to the development of this condition. These findings are consistent with those of a previous MR-based study using data from the Biobank in the UK, which also showed no causal association between moderate coffee consumption and BMD or the risk of bone fractures [11]. Therefore, the present study provided further support to this previous research. Some studies have indicated that regular consumption of green tea may prevent osteoporosis and fractures [30, 31]; however, there is no clear causal association between the two. In a recent study, researchers utilized MR to explore the association between tea consumption and the risk of osteoporosis. They performed a GWAS involving 349,376 European participants from the UK Biobank to examine the effects of genes related to tea consumption on the risk of developing osteoporosis [32]. Nevertheless, the association trend was unconfirmed in MR analysis, indicating that tea consumption may not have a direct impact on the risk of osteoporosis. These results are in line with findings of the present study.

The relationship between bitter non-alcoholic beverages, such as coffee and tea, and osteoporosis risk is complex and likely mediated by several biological mechanisms. For example, caffeine has been shown to reduce calcium absorption, which can lead to lower bone mineral density over time [33]. This study adds to existing evidence suggesting that while moderate coffee consumption may not significantly influence osteoporosis risk, high levels of caffeine intake, especially from multiple sources, could have detrimental effects on bone health.

Individuals consume a wide variety of beverages, thus, it is unclear whether drinking several bitter beverages over a long period of time could increase the risk of osteoporosis. The present study suggested that there could be a causal association between consumption of bitter non-alcoholic beverages and the risk of osteoporosis. Additional studies are needed to determine the exact correlation between the two. It was indicated that long-term consumption of bitter non-alcoholic beverages might have potential adverse effects on bone health, such as reducing bone density and increasing the risk of osteoporosis. One study found that consumption of caffeine-containing beverages that typically contain bitter compounds was associated with the lower bone density in European women [34]. Another study found an association between high alcohol intake and the increased fracture risk in both men and women [35]. However, the analysis of bitter alcoholic beverages was limited by the available SNP data, and a more detailed investigation into the relationship between different types of alcoholic beverages, such as beer, red wine, and white wine, and osteoporosis risk was not conducted in this study. Future research should address this gap by investigating the specific impact of various alcoholic beverages on bone health, as differences in alcohol content and other compounds may have distinct effects on osteoporosis risk. It is noteworthy that these studies did not necessarily prove causation and more research is required to fully understand the impact of bitter beverages on bone health. Regardless, it is generally advised to consume these beverages in moderation and include calcium-rich food in daily diet to promote bone health.

The present study had several strengths. Firstly, MR analysis was utilized that is relatively robust to confounding and reverse causality, and it could therefore provide strong evidence for causal association between lifestyle factors and the risk of osteoporosis. Secondly, smoking status was categorized into three groups and a thorough analysis was conducted to determine the association of each individual variable with osteoporosis and pathological fractures. This increased the accuracy and reliability of the findings. Thirdly, studies have been conducted on the causal association between coffee and alcohol consumption and the risk of osteoporosis [12, 13], while no study has examined the causal association between consumption of bitter beverages and the risk of osteoporosis and pathological fractures. This study is the first to investigate the potential association between the two factors.

This analysis also had some potential limitations. Firstly, the MR analysis utilized GWAS data solely from European populations, which might restrict the generalizability of the results to other populations. Although the GWAS data used in this study represented the largest population cohort available at the time, additional studies using data from other GWASs could provide a more comprehensive examination of the causal association between bitter beverage consumption and the risk of osteoporosis. Secondly, limitations in the analysis of SNPs for beer, red wine, and white wine in bitter alcohol beverages restricted the examination of the causal association between different alcohol types and the risk of osteoporosis. Therefore, further study addressing these limitations is warranted to provide more detailed findings. Furthermore, it is noteworthy that the present study did not utilize data from the UK Biobank, and the self-reported definition of osteoporosis in the UK Biobank might cause potential bias. Moreover, as osteoporosis is a silent disease, relying on self-reported data may not accurately reflect the actual prevalence of osteoporosis. Another limitation is the absence of variables for osteoporosis with pathological fractures, which could further limit the accuracy of self-reported data. Hence, it is imperative to further investigate the causal association between bitter beverage consumption and the risk of osteoporosis using data from various populations and different study designs to obtain more comprehensive and reliable findings.

In conclusion, this MR-based study provided genetic evidence supporting a positive causal association between smoking and the risk of osteoporosis, as well as osteoporosis with pathological fractures. Additionally, it revealed a positive causal association between the consumption of bitter beverages and the risk of osteoporosis. Therefore, it is crucial for individuals with a higher risk of developing osteoporosis to prioritize avoiding lifestyle factors, such as smoking, excessive consumption of bitter drinks, and other harmful behaviors. Due to the inconsistencies found among different populations and some limitations of the study, further research is required to more accurately characterize the specific relationship among smoking, consumption of bitter beverages, the risk of osteoporosis, and any resulting pathological fractures.

## Electronic supplementary material

Below is the link to the electronic supplementary material.

**Supplementary Material 1**: **Supplementary Table 1**. STROBE-MR checklist of recommended items to address in reports of Mendelian randomization studies. **Supplementary Table 2**. Detailed information on used studies. **Supplementary Table 3**. Detailed information on genetic instruments. **Supplementary Fig. 1**. Scatter plots of the 3MR modelsfor smoking per day that are causally related to osteoporosis. **Supplementary Fig. 2**. Scatter plots of the 3MR modelsfor bitter non-alcoholic beverages that are causally related to osteoporosis. **Supplementary Fig. 3**. Scatter plots of the 3MR modelsfor total bitter beverages that are causally related to osteoporosis. **Supplementary Fig. 4**. Scatter plots of the 3MR modelsfor lifetime smoking index that are causally related to osteoporosis with pathological fracture. **Supplementary Fig. 5**. The leave-one-out analysis of the causal association between smoking per day and the risk of osteoporosis. **Supplementary Fig. 6**. The leave-one-out analysis of the causal association between bitter non-alcoholic beverages consumption and the risk of osteoporosis. **Supplementary Fig. 7**. The leave-one-out analysis of the causal association between total bitter beverages consumption and the risk of osteoporosis. **Supplementary Fig. 8**. The leave-one-out analysis of the causal association between lifetime smoking index and the risk of osteoporosis with pathological fractures.

## Data Availability

The data that support the findings of this study are available from the corresponding author upon reasonable request.

## Abbreviations

IVW: Inverse-variance weighted
MR: Mendelian randomization
GWAS: Genome-wide association study
IV: Instrumental variable
RCTs: Randomized controlled trials
SNPs: Single-nucleotide polymorphisms
COPD: Chronic obstructive pulmonary disease
BMD: Bone mineral density
LD: Linkage disequilibrium
