# Supplementary material for: Causal association among smoking, bitter beverage consumption, and risk of osteoporosis: a two-sample mendelian randomization-based study
Source: Hereditas. 2025 Jan 24;162:7. doi: 10.1186/s41065-025-00371-1 (PMC11760116; doi:10.1186/s41065-025-00371-1)

Supplementary Table 1 STROBE-MR checklist of recommended items to address in reports of Mendelian randomization studies

| **Item No.** | **Section** | **Checklist item** | **pages** |
| --- | --- | --- | --- |
| 1 | **TITLE and ABSTRACT** | Indicate Mendelian randomization (MR) as the study’s design in the title and/or the abstract if that is a main purpose of the study | 1-2 |
|  | **INTRODUCTION** |  |  |
| 2 | **Background** | Explain the scientific background and rationale for the reported study. What is the exposure? Is a potential causal relationship between exposure and outcome plausible? Justify why MR is a helpful method to address the study question | 3-4 |
| 3 | **Objectives** | State specific objectives clearly, including pre-specified causal hypotheses (if any). State that MR is a method that, under specific assumptions, intends to estimate causal effects | 4 |
|  | **METHODS** |  |  |
| 4 | **Study design and data sources** | Present key elements of the study design early in the article. Consider including a table listing sources of data for all phases of the study. For each data source contributing to the analysis, describe the following: | 4-7 |
|  | a) | Setting: Describe the study design and the underlying population, if possible. Describe the setting, locations, and relevant dates, including periods of recruitment, exposure, follow-up, and data collection, when available. | 4 |
|  | b) | Participants: Give the eligibility criteria, and the sources and methods of selection of participants. Report the sample size, and whether any power or sample size calculations were carried out prior to the main analysis | 4-6 |
|  | c) | Describe measurement, quality control and selection of genetic variants | 6 |
|  | d) | For each exposure, outcome, and other relevant variables, describe methods of assessment and diagnostic criteria for diseases | 5-6 |
|  | e) | Provide details of ethics committee approval and participant informed consent, if relevant | 5-6 |
| 5 | **Assumptions** | Explicitly state the three core IV assumptions for the main analysis (relevance, independence and exclusion restriction) as well assumptions for any additional or sensitivity analysis | 4 |
| 6 | **Statistical methods: main analysis** | Describe statistical methods and statistics used | 6 |
|  | a) | Describe how quantitative variables were handled in the analyses (i.e., scale, units, model) | 6 |
|  | b) | Describe how genetic variants were handled in the analyses and, if applicable, how their weights were selected | 6 |
|  | c) | Describe the MR estimator (e.g. two-stage least squares, Wald ratio) and related statistics. Detail the included covariates and, in case of two-sample MR, whether the same covariate set was used for adjustment in the two samples | 6 |
|  | d) | Explain how missing data were addressed | 6 |
|  | e) | If applicable, indicate how multiple testing was addressed | 6-7 |

| 7 | **Assessment of assumptions** | Describe any methods or prior knowledge used to assess the assumptions or justify their validity | 6-7 |
| --- | --- | --- | --- |
| 8 | **Sensitivity analyses and additional analyses** | Describe any sensitivity analyses or additional analyses performed (e.g. comparison of effect estimates from different approaches, independent replication, bias analytic techniques, validation of instruments, simulations) | 7 |
| 9 | **Software and pre- registration** |  |  |
|  | a) | Name statistical software and package(s), including version and settings used | 6 |
|  | b) | State whether the study protocol and details were pre-registered (as well as when and where) | none |
|  | **RESULTS** |  |  |
| 10 | **Descriptive data** |  |  |
|  | a) | Report the numbers of individuals at each stage of included studies and reasons for exclusion. Consider use of a flow diagram | 7 |
|  | b) | Report summary statistics for phenotypic exposure(s), outcome(s), and other relevant variables (e.g. means, SDs, proportions) | 7-9 |
|  | c) | If the data sources include meta-analyses of previous studies, provide the assessments of heterogeneity across these studies | 7-9 |
|  | d) | For two-sample MR:   1. Provide justification of the similarity of the genetic variant-exposure associations between the exposure and outcome samples 2. Provide information on the number of individuals who overlap between the exposure and outcome studies | 7-9 |
| 11 | **Main results** |  |  |
|  | a) | Report the associations between genetic variant and exposure, and between genetic variant and outcome, preferably on an interpretable scale | 7-9 |
|  | b) | Report MR estimates of the relationship between exposure and outcome, and the measures of uncertainty from the MR analysis, on an interpretable scale, such as odds ratio or relative risk per SD difference | 7-9 |
|  | c) | If relevant, consider translating estimates of relative risk into absolute risk for a meaningful time period | 7-9 |
|  | d) | Consider plots to visualize results (e.g. forest plot, scatterplot of associations between genetic variants and outcome versus between genetic variants and exposure) | 7-9 |
| 12 | **Assessment of assumptions** |  |  |
|  | a) | Report the assessment of the validity of the assumptions | 8-9 |
|  | b) | Report any additional statistics (e.g., assessments of heterogeneity across genetic variants, such as *I^2^*, Q statistic or E-value) | 7-9 |
| 13 | **Sensitivity analyses**  **and additional analyses** |  |  |

|  | a) | Report any sensitivity analyses to assess the robustness of the main results to violations of the assumptions | 7-9 |
| --- | --- | --- | --- |
|  | b) | Report results from other sensitivity analyses or additional analyses | 7-9 |
|  | c) | Report any assessment of direction of causal relationship (e.g., bidirectional MR) | 7-9 |
|  | d) | When relevant, report and compare with estimates from non-MR analyses | 7-9 |
|  | e) | Consider additional plots to visualize results (e.g., leave-one-out analyses) | 7-9 |
|  | **DISCUSSION** |  |  |
| 14 | **Key results** | Summarize key results with reference to study objectives | 9-12 |
| 15 | **Limitations** | Discuss limitations of the study, taking into account the validity of the IV assumptions, other sources of potential bias, and imprecision. Discuss both direction and magnitude of any potential bias and any efforts to address them | 12 |
| 16 | **Interpretation** |  |  |
|  | a) | Meaning: Give a cautious overall interpretation of results in the context of their limitations and in comparison with other studies | 9-12 |
|  | b) | Mechanism: Discuss underlying biological mechanisms that could drive a potential causal relationship between the investigated exposure and the outcome, and whether the gene-environment equivalence assumption is reasonable. Use causal language carefully, clarifying that IV estimates may provide causal effects only under certain assumptions | 9-12 |
|  | c) | Clinical relevance: Discuss whether the results have clinical or public policy relevance, and to what extent they inform effect sizes of possible interventions | 12 |
| 17 | **Generalizability** | Discuss the generalizability of the study results (a) to other populations, (b) across other exposure periods/timings, and (c) across other levels of exposure | 12 |
|  | **OTHER INFORMATION** |  |  |
| 18 | **Funding** | Describe sources of funding and the role of funders in the present study and, if applicable, sources of funding for the databases and original study or studies on which the present study is based | 13 |
| 19 | **Data and data sharing** | Provide the data used to perform all analyses or report where and how the data can be accessed, and reference these sources in the article. Provide the statistical code needed to reproduce the results in the article, or report whether the code is publicly accessible and if so, where | 13 |
| 20 | **Conflicts of Interest** | All authors should declare all potential conflicts of interest | 13 |

This checklist is copyrighted by the Equator Network under the Creative Commons Attribution 3.0 Unported (CC BY 3.0) license.

1. Skrivankova VW, Richmond RC, Woolf BAR, Yarmolinsky J, Davies NM, Swanson SA, et al. Strengthening the Reporting of Observational Studies in Epidemiology using Mendelian Randomization (STROBE-MR) Statement. JAMA. 2021;under review.
2. Skrivankova VW, Richmond RC, Woolf BAR, Davies NM, Swanson SA, VanderWeele TJ, et al. Strengthening the Reporting of Observational Studies in Epidemiology using Mendelian Randomisation (STROBE-MR): Explanation and Elaboration. BMJ. 2021;375:n2233.

Supplementary Table 2. Detailed information on the included studies

| **Exposure or outcome** | **Definition** | **Unit** | **Participants** **included in analysis** | **Adjustments** | **Identified SNPs** | **PubMed ID/Web source** |
| --- | --- | --- | --- | --- | --- | --- |
| **Smoking** |  |  |  |  |  |  |
| Smoking initiation | probability of ever smoked regularly | SD in prevalence of smoking initiation | 1232,091 European-descent individuals | age, sex, and the first ten genetic principal components | 301 | 36309747 |
| Smoking per day | average number of cigarettes smoked per day both in current smoker and former smoker | SD increase of log-transformed alcoholic smoking/ day | 216,590 European-descent individuals | age, sex, and the first 10 genetic principal components | 17 | 36309747 |
| Lifetime smoking index | takes into account smoking status as well as smoking duration, heaviness, and cessation in ever smokers | SD increase of lifetime smoking index | 462,690 European-descent individuals | genotyping chip and sex | 108 | 36309747 |
| **Bitter beverage consumption** |  |  |  |  |  |  |
| Total bitter beverages | self-reported consumption of bitter beverages were collected using a 24 h recall questionnaire, included coffee, tea,  grapefruit juice and bitter tasting alcoholic beverages (beer/cider,  red wine and liquor) | servings/day | 125,776 European-descent individuals | age, sex, total energy, proportion of 24-h recalls with reporting typical intake, and top 20 principal components of population sub-structure | 3 | 31046077 |
| Bitter alcoholic beverages | self-reported consumption of alcohol was collected using a 24 h recall questionnaire, participants were asked about their alcohol  drinking status (never, former, current, unknown), frequency  (weekly or monthly) and types (e.g. red wine, white wine, beer/-  cider, spirit) | servings/day | 376,372 European-descent individuals | age, sex | 7 | 31046077 |
| Bitter nonalcoholic beverages | self-reported consumption of nonalcoholic beverage was collected using a 24 h recall questionnaire | servings/day | 122,435 European-descent individuals | age, sex, total energy, proportion of 24-h recalls with reporting typical intake, and top 20 principal components of population sub-structure | 4 | 31046077 |
| Tea | self-reported consumption of tea was collected using a 24 h recall questionnaire,total tea consumption was based on the question ‘How many cups of tea do you drink each day ? | servings/day | 376,822 European-descent individuals | age, sex | 13 | 31046077 |
| Coffee | self-reported consumption of coffee was collected using a 24 h recall questionnaire ,total coffee consumption was based on the question ‘How many cups of coffee do you drink each day (include decaffeinated coffee)? | servings/day | 376,923 European-descent individuals | age, sex | 32 | 31046077 |
| **Outcome** |  |  |  |  |  |  |
| Osteoporosis | M13_OSTEOPOROSIS | - | 6,303cases and 325,717 non-cases | - | 20,169,250 | FinnGen consortium (https://www.finngen.fi/fi) |
| Osteoporosis with pathological fracture | OSTEOPOROSIS_FRACTURE_FG | - | 1,433cases and 261,098 non-cases | - | 20,166,569 | FinnGen consortium (https://www.finngen.fi/fi) |

SD, Standard deviation;

Here, one serving of a beverage can be 1 cup (e.g. coffee, tea), 1 medium glass (e.g. fruit juice, wine), 1 shot/measure (e.g. liquor/spirit) or 1 pint/bottle/can

(e.g. beer, carbonated beverages).

Supplementary Table 3. Detailed information on genetic instruments

| **Exposure** | **SNP** | **EA** | **OEA** | **EAF** | **BETA** | **SE** | **P-value** |
| --- | --- | --- | --- | --- | --- | --- | --- |
| Smoking per day | rs2118359 | T | G | 0.229 | 0.026 | 0.005 | 1.03E-08 |
| Smoking per day | rs78385743 | T | A | 0.034 | -0.062 | 0.01 | 1.36E-10 |
| Smoking per day | rs12442456 | T | G | 0.148 | -0.06 | 0.006 | 2.45E-27 |
| Smoking per day | rs114642875 | A | G | 0.015 | 0.083 | 0.014 | 6.61E-09 |
| Smoking per day | rs9788721 | T | C | 0.652 | -0.09 | 0.004 | ###### |
| Smoking per day | rs147760547 | G | A | 0.022 | 0.079 | 0.012 | 2.22E-10 |
| Smoking per day | rs189302121 | T | A | 0.028 | 0.06 | 0.01 | 7.53E-09 |
| Smoking per day | rs112272197 | T | C | 0.04 | 0.064 | 0.01 | 3.69E-11 |
| Smoking per day | rs3813567 | A | G | 0.779 | 0.058 | 0.005 | 2.58E-35 |
| Smoking per day | rs117189732 | T | C | 0.083 | 0.047 | 0.007 | 2.35E-12 |
| Smoking per day | rs8034274 | C | T | 0.565 | -0.041 | 0.004 | 1.40E-26 |
| Smoking per day | rs79409323 | C | T | 0.021 | -0.06 | 0.011 | 4.56E-08 |
| Smoking per day | rs1579233 | G | A | 0.571 | -0.023 | 0.004 | 1.94E-09 |
| Smoking per day | rs56113850 | C | T | 0.568 | 0.04 | 0.004 | 8.58E-26 |
| Smoking per day | rs28399442 | A | C | 0.02 | -0.098 | 0.011 | 4.22E-18 |
| Smoking per day | rs1801272 | T | A | 0.023 | -0.085 | 0.013 | 3.24E-11 |
| Smoking per day | rs59586387 | G | C | 0.072 | -0.039 | 0.007 | 2.27E-08 |
| Smoking per day | rs2273500 | C | T | 0.159 | 0.034 | 0.005 | 8.58E-11 |
| Smoking per day | rs215600 | A | G | 0.64 | -0.025 | 0.004 | 2.06E-10 |
| Smoking per day | rs58379124 | C | T | 0.748 | 0.034 | 0.004 | 2.06E-14 |
| Smoking per day | rs3025383 | C | T | 0.18 | -0.032 | 0.005 | 1.19E-11 |
| Lifetime smoking index | rs9435340 | T | A | 0.344 | 0.008 | 0.001 | 1.20E-08 |
| Lifetime smoking index | rs10918701 | G | A | 0.372 | 0.008 | 0.001 | 2.10E-08 |
| Lifetime smoking index | rs4949465 | T | C | 0.87 | -0.012 | 0.002 | 1.70E-08 |
| Lifetime smoking index | rs549845 | G | A | 0.301 | 0.011 | 0.002 | 8.30E-14 |
| Lifetime smoking index | rs1933270 | T | G | 0.364 | 0.009 | 0.001 | 1.50E-10 |
| Lifetime smoking index | rs7528604 | G | A | 0.566 | 0.01 | 0.001 | 5.70E-12 |
| Lifetime smoking index | rs11210229 | A | G | 0.384 | 0.012 | 0.001 | 2.00E-16 |
| Lifetime smoking index | rs7553348 | G | A | 0.438 | 0.01 | 0.001 | 5.20E-12 |
| Lifetime smoking index | rs1193237 | G | C | 0.439 | -0.008 | 0.001 | 2.80E-08 |
| Lifetime smoking index | rs10922907 | A | T | 0.451 | 0.01 | 0.001 | 3.00E-13 |
| Lifetime smoking index | rs1931263 | G | T | 0.51 | -0.008 | 0.001 | 4.00E-08 |
| Lifetime smoking index | rs7519626 | C | T | 0.324 | 0.008 | 0.001 | 1.20E-08 |
| Lifetime smoking index | rs7077678 | C | T | 0.623 | 0.009 | 0.001 | 2.60E-09 |
| Lifetime smoking index | rs12244388 | G | A | 0.661 | -0.013 | 0.001 | 1.40E-19 |
| Lifetime smoking index | rs3896224 | A | G | 0.585 | 0.01 | 0.001 | 1.10E-11 |
| Lifetime smoking index | rs2675638 | G | A | 0.581 | 0.008 | 0.001 | 1.30E-09 |
| Lifetime smoking index | rs10823968 | A | T | 0.633 | 0.008 | 0.001 | 2.10E-08 |
| Lifetime smoking index | rs11255908 | T | G | 0.743 | -0.01 | 0.002 | 2.30E-10 |
| Lifetime smoking index | rs17553262 | A | C | 0.885 | -0.013 | 0.002 | 5.30E-09 |
| Lifetime smoking index | rs9919670 | G | A | 0.612 | -0.015 | 0.001 | 7.60E-27 |
| Lifetime smoking index | rs34866095 | A | G | 0.686 | -0.009 | 0.002 | 1.20E-08 |
| Lifetime smoking index | rs75742406 | G | A | 0.739 | 0.01 | 0.002 | 1.30E-09 |
| Lifetime smoking index | rs17309874 | G | A | 0.74 | -0.011 | 0.002 | 9.70E-13 |
| Lifetime smoking index | rs4391802 | A | G | 0.707 | 0.01 | 0.002 | 1.40E-11 |
| Lifetime smoking index | rs112282219 | G | A | 0.959 | -0.023 | 0.004 | 3.80E-11 |
| Lifetime smoking index | rs74086911 | G | A | 0.925 | 0.015 | 0.003 | 2.10E-08 |
| Lifetime smoking index | rs7297175 | T | C | 0.431 | -0.008 | 0.001 | 6.60E-09 |
| Lifetime smoking index | rs10879871 | T | G | 0.343 | -0.01 | 0.001 | 5.00E-11 |
| Lifetime smoking index | rs12831617 | C | T | 0.764 | -0.009 | 0.002 | 1.90E-08 |
| Lifetime smoking index | rs7333559 | G | A | 0.212 | 0.011 | 0.002 | 3.20E-10 |
| Lifetime smoking index | rs6562474 | C | G | 0.651 | 0.008 | 0.001 | 1.00E-08 |
| Lifetime smoking index | rs3742365 | T | C | 0.595 | -0.011 | 0.001 | 2.50E-14 |
| Lifetime smoking index | rs860326 | C | T | 0.428 | 0.008 | 0.001 | 2.70E-09 |
| Lifetime smoking index | rs7155595 | A | C | 0.674 | -0.009 | 0.001 | 2.50E-09 |
| Lifetime smoking index | rs35175834 | G | A | 0.788 | -0.016 | 0.002 | 4.60E-22 |
| Lifetime smoking index | rs28485305 | C | T | 0.631 | 0.008 | 0.001 | 2.60E-08 |
| Lifetime smoking index | rs8042849 | C | T | 0.342 | 0.019 | 0.001 | 1.80E-39 |
| Lifetime smoking index | rs8042134 | T | G | 0.541 | -0.01 | 0.001 | 1.30E-12 |
| Lifetime smoking index | rs6598539 | T | C | 0.489 | -0.008 | 0.001 | 4.50E-09 |
| Lifetime smoking index | rs12708665 | A | G | 0.285 | -0.009 | 0.002 | 3.50E-09 |
| Lifetime smoking index | rs57611503 | G | A | 0.485 | 0.008 | 0.001 | 4.00E-08 |
| Lifetime smoking index | rs889398 | C | T | 0.588 | 0.009 | 0.001 | 6.30E-11 |
| Lifetime smoking index | rs11861214 | G | T | 0.784 | 0.009 | 0.002 | 2.00E-08 |
| Lifetime smoking index | rs60952428 | T | C | 0.909 | 0.013 | 0.002 | 3.00E-08 |
| Lifetime smoking index | rs1050847 | C | T | 0.426 | 0.008 | 0.001 | 1.40E-08 |
| Lifetime smoking index | rs369230 | G | T | 0.308 | -0.009 | 0.002 | 1.80E-09 |
| Lifetime smoking index | rs8614 | C | A | 0.817 | -0.011 | 0.002 | 1.80E-10 |
| Lifetime smoking index | rs732083 | G | A | 0.333 | 0.008 | 0.001 | 1.50E-08 |
| Lifetime smoking index | rs9904288 | T | C | 0.708 | 0.008 | 0.002 | 3.10E-08 |
| Lifetime smoking index | rs67596067 | G | A | 0.649 | -0.009 | 0.001 | 1.20E-09 |
| Lifetime smoking index | rs12967855 | A | G | 0.331 | 0.008 | 0.001 | 3.10E-08 |
| Lifetime smoking index | rs62098013 | G | A | 0.64 | -0.009 | 0.001 | 4.10E-09 |
| Lifetime smoking index | rs71367545 | G | A | 0.791 | -0.01 | 0.002 | 1.40E-09 |
| Lifetime smoking index | rs35343344 | C | A | 0.733 | 0.009 | 0.002 | 8.80E-09 |
| Lifetime smoking index | rs76608582 | C | A | 0.953 | 0.022 | 0.003 | 3.20E-10 |
| Lifetime smoking index | rs2678670 | A | T | 0.486 | 0.009 | 0.001 | 3.10E-10 |
| Lifetime smoking index | rs62155874 | A | G | 0.873 | -0.017 | 0.002 | 5.20E-16 |
| Lifetime smoking index | rs3811038 | T | C | 0.724 | -0.01 | 0.002 | 8.90E-10 |
| Lifetime smoking index | rs2890772 | G | T | 0.413 | -0.014 | 0.001 | 2.10E-22 |
| Lifetime smoking index | rs62175972 | T | C | 0.966 | 0.022 | 0.004 | 1.70E-08 |
| Lifetime smoking index | rs3769949 | T | A | 0.528 | -0.008 | 0.001 | 2.50E-09 |
| Lifetime smoking index | rs13009008 | A | G | 0.328 | 0.009 | 0.001 | 4.60E-09 |
| Lifetime smoking index | rs4473348 | A | T | 0.25 | -0.01 | 0.002 | 6.40E-11 |
| Lifetime smoking index | rs12623702 | A | G | 0.613 | -0.01 | 0.001 | 7.70E-12 |
| Lifetime smoking index | rs6741228 | T | C | 0.433 | 0.008 | 0.001 | 1.60E-08 |
| Lifetime smoking index | rs62135536 | C | T | 0.968 | 0.024 | 0.004 | 8.00E-10 |
| Lifetime smoking index | rs7569203 | A | C | 0.689 | -0.011 | 0.002 | 7.40E-13 |
| Lifetime smoking index | rs13016665 | C | A | 0.577 | -0.008 | 0.001 | 1.80E-09 |
| Lifetime smoking index | rs4671357 | T | C | 0.519 | -0.009 | 0.001 | 1.10E-11 |
| Lifetime smoking index | rs359243 | T | C | 0.393 | -0.009 | 0.001 | 9.50E-10 |
| Lifetime smoking index | rs2867112 | T | G | 0.835 | 0.015 | 0.002 | 4.80E-15 |
| Lifetime smoking index | rs4814873 | C | T | 0.767 | 0.01 | 0.002 | 2.90E-09 |
| Lifetime smoking index | rs6119897 | G | A | 0.762 | -0.013 | 0.002 | 3.60E-15 |
| Lifetime smoking index | rs12481282 | G | C | 0.722 | -0.009 | 0.002 | 7.80E-09 |
| Lifetime smoking index | rs348809 | A | G | 0.348 | -0.008 | 0.001 | 1.30E-08 |
| Lifetime smoking index | rs6011779 | C | T | 0.191 | 0.019 | 0.002 | 2.30E-27 |
| Lifetime smoking index | rs147412694 | G | A | 0.85 | -0.012 | 0.002 | 2.90E-09 |
| Lifetime smoking index | rs2838834 | C | T | 0.699 | -0.009 | 0.002 | 6.30E-10 |
| Lifetime smoking index | rs136233 | A | G | 0.809 | -0.01 | 0.002 | 1.80E-08 |
| Lifetime smoking index | rs202645 | A | G | 0.203 | -0.01 | 0.002 | 3.90E-09 |
| Lifetime smoking index | rs326341 | G | A | 0.525 | 0.009 | 0.001 | 1.20E-11 |
| Lifetime smoking index | rs73220544 | A | C | 0.842 | -0.011 | 0.002 | 1.50E-08 |
| Lifetime smoking index | rs9842947 | C | T | 0.326 | -0.009 | 0.001 | 3.10E-09 |
| Lifetime smoking index | rs6779302 | G | T | 0.633 | -0.009 | 0.001 | 1.20E-09 |
| Lifetime smoking index | rs6778080 | T | C | 0.267 | 0.011 | 0.002 | 1.30E-12 |
| Lifetime smoking index | rs775758 | A | T | 0.433 | 0.008 | 0.001 | 1.10E-08 |
| Lifetime smoking index | rs421983 | T | C | 0.519 | 0.009 | 0.001 | 3.30E-10 |
| Lifetime smoking index | rs72678864 | G | A | 0.829 | 0.012 | 0.002 | 1.60E-11 |
| Lifetime smoking index | rs17576594 | G | A | 0.724 | 0.011 | 0.002 | 1.70E-12 |
| Lifetime smoking index | rs61796681 | A | T | 0.912 | -0.013 | 0.002 | 4.20E-08 |
| Lifetime smoking index | rs624833 | T | G | 0.695 | 0.009 | 0.002 | 6.60E-10 |
| Lifetime smoking index | rs317021 | T | A | 0.814 | -0.012 | 0.002 | 1.10E-10 |
| Lifetime smoking index | rs4957528 | A | C | 0.208 | -0.01 | 0.002 | 4.20E-09 |
| Lifetime smoking index | rs11948770 | T | C | 0.768 | -0.01 | 0.002 | 4.90E-10 |
| Lifetime smoking index | rs329120 | C | T | 0.581 | 0.01 | 0.001 | 6.30E-12 |
| Lifetime smoking index | rs986391 | G | A | 0.367 | 0.011 | 0.001 | 9.40E-15 |
| Lifetime smoking index | rs13153393 | A | G | 0.884 | -0.014 | 0.002 | 2.50E-10 |
| Lifetime smoking index | rs245774 | A | G | 0.272 | -0.009 | 0.002 | 7.40E-09 |
| Lifetime smoking index | rs71627581 | G | A | 0.889 | 0.013 | 0.002 | 1.60E-09 |
| Lifetime smoking index | rs10052591 | T | C | 0.573 | 0.008 | 0.001 | 2.10E-09 |
| Lifetime smoking index | rs2080870 | A | T | 0.258 | 0.009 | 0.002 | 4.90E-08 |
| Lifetime smoking index | rs4571506 | C | T | 0.54 | 0.008 | 0.001 | 1.50E-08 |
| Lifetime smoking index | rs7766610 | C | A | 0.183 | 0.013 | 0.002 | 2.20E-12 |
| Lifetime smoking index | rs6935954 | A | G | 0.421 | 0.01 | 0.001 | 8.20E-12 |
| Lifetime smoking index | rs2254710 | C | A | 0.236 | 0.009 | 0.002 | 3.50E-08 |
| Lifetime smoking index | rs2894808 | T | A | 0.922 | -0.015 | 0.003 | 3.50E-09 |
| Lifetime smoking index | rs12202536 | A | G | 0.513 | -0.008 | 0.001 | 2.80E-09 |
| Lifetime smoking index | rs10282292 | C | T | 0.362 | 0.009 | 0.001 | 5.90E-10 |
| Lifetime smoking index | rs2401924 | G | C | 0.502 | 0.011 | 0.001 | 2.70E-14 |
| Lifetime smoking index | rs7807019 | A | G | 0.54 | -0.01 | 0.001 | 6.70E-14 |
| Lifetime smoking index | rs6957896 | C | T | 0.503 | -0.008 | 0.001 | 4.50E-08 |
| Lifetime smoking index | rs4731925 | C | T | 0.316 | -0.008 | 0.001 | 2.60E-08 |
| Lifetime smoking index | rs10226228 | A | G | 0.63 | -0.011 | 0.001 | 2.00E-15 |
| Lifetime smoking index | rs1922018 | C | T | 0.364 | 0.01 | 0.001 | 3.00E-12 |
| Lifetime smoking index | rs11768481 | C | A | 0.666 | 0.009 | 0.001 | 9.90E-10 |
| Lifetime smoking index | rs6962772 | A | G | 0.846 | 0.011 | 0.002 | 7.80E-09 |
| Lifetime smoking index | rs11783093 | C | T | 0.839 | 0.016 | 0.002 | 1.20E-16 |
| Lifetime smoking index | rs2062882 | G | A | 0.587 | -0.008 | 0.001 | 1.10E-08 |
| Lifetime smoking index | rs72674867 | A | T | 0.765 | 0.009 | 0.002 | 3.80E-08 |
| Lifetime smoking index | rs35169606 | T | G | 0.612 | 0.009 | 0.001 | 1.20E-09 |
| Lifetime smoking index | rs1221148 | C | G | 0.587 | 0.009 | 0.001 | 7.30E-11 |
| Lifetime smoking index | rs13296519 | G | T | 0.606 | -0.01 | 0.001 | 8.10E-12 |
| Lifetime smoking index | rs113382419 | C | A | 0.889 | -0.028 | 0.002 | 3.00E-37 |
| Lifetime smoking index | rs4543592 | T | C | 0.52 | -0.009 | 0.001 | 4.50E-10 |
| Lifetime smoking index | rs7039819 | G | A | 0.427 | 0.009 | 0.001 | 5.10E-10 |
| Lifetime smoking index | rs1246265 | T | C | 0.305 | -0.009 | 0.002 | 4.20E-09 |
| Smoking Initiation | rs12027999 | C | T | 0.12 | -0.0143 | 0.00244 | 5.33E-10 |
| Smoking Initiation | rs45444697 | G | C | 0.212 | 0.00955 | 0.00151 | 2.72E-10 |
| Smoking Initiation | rs2901785 | A | G | 0.446 | -0.00816 | 0.00161 | 1.47E-11 |
| Smoking Initiation | rs147052174 | T | G | 0.0171 | 0.0368 | 0.00631 | 2.30E-10 |
| Smoking Initiation | rs3820277 | T | G | 0.526 | -0.0098 | 0.00162 | 1.57E-13 |
| Smoking Initiation | rs35656245 | A | G | 0.276 | 0.00925 | 0.00179 | 2.23E-08 |
| Smoking Initiation | rs12739243 | C | T | 0.221 | -0.0122 | 0.00192 | 4.45E-12 |
| Smoking Initiation | rs12563365 | A | G | 0.556 | 0.00927 | 0.00162 | 1.05E-10 |
| Smoking Initiation | rs876793 | C | T | 0.349262 | -0.00861 | 0.001315 | 5.69E-11 |
| Smoking Initiation | rs1889571 | G | T | 0.131 | 0.0132 | 0.00236 | 4.19E-09 |
| Smoking Initiation | rs10914684 | A | G | 0.324 | -0.00836 | 0.00172 | 6.32E-09 |
| Smoking Initiation | rs2637869 | A | G | 0.297 | 0.00893 | 0.00174 | 6.54E-11 |
| Smoking Initiation | rs12755632 | G | A | 0.316 | -0.00798 | 0.00172 | 1.93E-08 |
| Smoking Initiation | rs951740 | A | G | 0.625 | 0.0163 | 0.00166 | 3.82E-29 |
| Smoking Initiation | rs925524 | G | A | 0.71 | 0.00814 | 0.00176 | 2.94E-08 |
| Smoking Initiation | rs12022778 | C | A | 0.202 | 0.015 | 0.00198 | 3.18E-17 |
| Smoking Initiation | rs11587399 | T | A | 0.221 | -0.00858 | 0.00148 | 7.25E-09 |
| Smoking Initiation | rs4912332 | T | C | 0.491 | 0.00845 | 0.0016 | 2.94E-08 |
| Smoking Initiation | rs1937443 | G | C | 0.563 | 0.0129 | 0.00162 | 1.79E-15 |
| Smoking Initiation | rs1022528 | A | G | 0.344 | 0.00837 | 0.00129 | 8.48E-11 |
| Smoking Initiation | rs12740789 | A | G | 0.178 | -0.0137 | 0.001597 | 1.18E-17 |
| Smoking Initiation | rs80054503 | C | T | 0.116 | -0.0128 | 0.002162 | 3.10E-09 |
| Smoking Initiation | rs10789369 | G | A | 0.615 | -0.013 | 0.00165 | 3.39E-19 |
| Smoking Initiation | rs1514176 | A | G | 0.58 | -0.0107 | 0.00162 | 7.67E-14 |
| Smoking Initiation | rs10873871 | G | A | 0.207 | 0.0098 | 0.00199 | 2.82E-08 |
| Smoking Initiation | rs12130857 | A | G | 0.324968 | -0.00867 | 0.001309 | 3.65E-11 |
| Smoking Initiation | rs301807 | G | A | 0.57 | 0.0112 | 0.00163 | 2.50E-12 |
| Smoking Initiation | rs11162019 | T | C | 0.363 | -0.00932 | 0.00166 | 5.06E-09 |
| Smoking Initiation | rs1008078 | T | C | 0.402 | 0.0119 | 0.00164 | 1.63E-18 |
| Smoking Initiation | rs1935571 | G | T | 0.48 | -0.0104 | 0.0016 | 6.99E-10 |
| Smoking Initiation | rs7920501 | A | T | 0.465 | -0.00911 | 0.0016 | 1.25E-09 |
| Smoking Initiation | rs7901883 | A | G | 0.230322 | -0.00934 | 0.001467 | 1.98E-10 |
| Smoking Initiation | rs11594623 | C | T | 0.234241 | 0.0132 | 0.00144 | 7.45E-20 |
| Smoking Initiation | rs11191269 | G | C | 0.193294 | 0.00855 | 0.00156 | 4.61E-08 |
| Smoking Initiation | rs28408682 | G | A | 0.600019 | 0.00805 | 0.001255 | 1.41E-10 |
| Smoking Initiation | rs12244388 | A | G | 0.35 | 0.0137 | 0.00169 | 4.31E-22 |
| Smoking Initiation | rs111842178 | G | A | 0.231008 | 0.0129 | 0.001841 | 2.24E-12 |
| Smoking Initiation | rs34970111 | T | C | 0.458 | -0.00718 | 0.001262 | 1.28E-08 |
| Smoking Initiation | rs9787523 | C | T | 0.418 | -0.00883 | 0.00165 | 1.42E-09 |
| Smoking Initiation | rs11192347 | A | G | 0.104 | -0.0129 | 0.002084 | 6.15E-10 |
| Smoking Initiation | rs1291821 | G | A | 0.534 | 0.00807 | 0.00161 | 1.39E-08 |
| Smoking Initiation | rs10885480 | C | T | 0.284 | -0.00953 | 0.00178 | 3.83E-11 |
| Smoking Initiation | rs4752018 | A | C | 0.231 | 0.0107 | 0.0019 | 4.42E-10 |
| Smoking Initiation | rs9423279 | G | C | 0.645 | -0.0102 | 0.00183 | 3.06E-12 |
| Smoking Initiation | rs11258417 | T | C | 0.391 | -0.00852 | 0.00163 | 2.71E-08 |
| Smoking Initiation | rs7072776 | G | A | 0.712 | -0.0138 | 0.00179 | 5.66E-15 |
| Smoking Initiation | rs2796793 | A | G | 0.452 | 0.0082 | 0.00161 | 1.55E-08 |
| Smoking Initiation | rs1733760 | C | T | 0.51 | 0.00821 | 0.00161 | 6.70E-09 |
| Smoking Initiation | rs7921378 | C | G | 0.482 | -0.0142 | 0.00161 | 6.10E-20 |
| Smoking Initiation | rs10905461 | C | T | 0.748 | -0.0104 | 0.00186 | 2.36E-08 |
| Smoking Initiation | rs76460663 | G | C | 0.041056 | -0.0226 | 0.003426 | 4.15E-11 |
| Smoking Initiation | rs2155646 | C | T | 0.4 | 0.021 | 0.00163 | 9.44E-48 |
| Smoking Initiation | rs1713676 | G | A | 0.522512 | -0.00802 | 0.00122 | 5.38E-11 |
| Smoking Initiation | rs238896 | A | G | 0.49 | -0.00989 | 0.00162 | 3.65E-11 |
| Smoking Initiation | rs540860 | G | A | 0.543 | 0.0101 | 0.00161 | 5.75E-12 |
| Smoking Initiation | rs1944689 | T | G | 0.785911 | 0.00859 | 0.00151 | 1.27E-08 |
| Smoking Initiation | rs1834306 | G | A | 0.579399 | -0.00711 | 0.00127 | 1.96E-08 |
| Smoking Initiation | rs1106363 | T | C | 0.344579 | 0.00833 | 0.00129 | 9.20E-11 |
| Smoking Initiation | rs2010921 | A | G | 0.311 | 0.00897 | 0.00174 | 2.47E-10 |
| Smoking Initiation | rs6265 | T | C | 0.188 | -0.0165 | 0.00204 | 2.81E-19 |
| Smoking Initiation | rs4275621 | G | A | 0.382 | -0.0103 | 0.00126 | 3.76E-16 |
| Smoking Initiation | rs62618693 | T | C | 0.0428 | -0.0204 | 0.00415 | 2.09E-08 |
| Smoking Initiation | rs2939756 | A | G | 0.48 | -0.00908 | 0.00161 | 7.45E-10 |
| Smoking Initiation | rs1381775 | C | T | 0.712 | -0.00754 | 0.00177 | 2.79E-08 |
| Smoking Initiation | rs2959084 | A | G | 0.704674 | 0.00817 | 0.00134 | 9.82E-10 |
| Smoking Initiation | rs3740977 | C | T | 0.167 | 0.0103 | 0.00216 | 1.17E-08 |
| Smoking Initiation | rs61886926 | T | C | 0.384 | -0.00987 | 0.00163 | 7.30E-12 |
| Smoking Initiation | rs61884449 | T | C | 0.149183 | 0.00962 | 0.001722 | 2.32E-08 |
| Smoking Initiation | rs644740 | T | C | 0.457 | -0.00839 | 0.00161 | 3.67E-08 |
| Smoking Initiation | rs7943721 | A | G | 0.829 | -0.0117 | 0.00218 | 3.58E-10 |
| Smoking Initiation | rs7929518 | G | A | 0.773 | 0.0119 | 0.00192 | 2.55E-10 |
| Smoking Initiation | rs586699 | A | G | 0.543 | -0.0078 | 0.0016 | 7.29E-09 |
| Smoking Initiation | rs77215829 | C | A | 0.131 | -0.013 | 0.00243 | 2.02E-10 |
| Smoking Initiation | rs1109480 | A | G | 0.384 | -0.0101 | 0.00167 | 1.84E-10 |
| Smoking Initiation | rs11611651 | A | G | 0.0868 | 0.0141 | 0.0028 | 2.05E-09 |
| Smoking Initiation | rs11057005 | G | A | 0.441 | -0.0104 | 0.00163 | 9.12E-10 |
| Smoking Initiation | rs13906 | T | C | 0.109 | -0.0118 | 0.00253 | 1.98E-09 |
| Smoking Initiation | rs4759229 | G | A | 0.656 | 0.01 | 0.00168 | 6.53E-09 |
| Smoking Initiation | rs7969559 | G | A | 0.713 | -0.0097 | 0.00178 | 1.53E-09 |
| Smoking Initiation | rs7134009 | C | T | 0.287 | -0.00904 | 0.00178 | 4.30E-08 |
| Smoking Initiation | rs7333559 | A | G | 0.783 | -0.0127 | 0.002 | 5.94E-14 |
| Smoking Initiation | rs1108130 | A | T | 0.212 | 0.0116 | 0.00151 | 1.57E-14 |
| Smoking Initiation | rs12855717 | T | C | 0.538 | 0.00753 | 0.00124 | 1.22E-09 |
| Smoking Initiation | rs17197663 | A | G | 0.125 | -0.0117 | 0.00245 | 2.06E-08 |
| Smoking Initiation | rs4264267 | T | C | 0.527042 | 0.00713 | 0.00123 | 6.82E-09 |
| Smoking Initiation | rs61959481 | A | G | 0.21 | -0.0113 | 0.00203 | 7.95E-11 |
| Smoking Initiation | rs3098272 | C | A | 0.798794 | -0.00867 | 0.001547 | 2.08E-08 |
| Smoking Initiation | rs9538162 | C | T | 0.415874 | 0.0085 | 0.001263 | 1.76E-11 |
| Smoking Initiation | rs1413119 | T | C | 0.396318 | -0.00734 | 0.001254 | 4.77E-09 |
| Smoking Initiation | rs56367474 | T | C | 0.304 | -0.00873 | 0.00175 | 4.20E-10 |
| Smoking Initiation | rs55786907 | G | A | 0.162492 | 0.00931 | 0.001654 | 1.84E-08 |
| Smoking Initiation | rs4886207 | C | T | 0.637 | -0.00872 | 0.00166 | 8.78E-10 |
| Smoking Initiation | rs9540731 | T | C | 0.509 | -0.0094 | 0.0016 | 3.42E-12 |
| Smoking Initiation | rs9545155 | C | T | 0.478 | -0.00889 | 0.0016 | 3.04E-10 |
| Smoking Initiation | rs1772572 | A | C | 0.324132 | -0.00807 | 0.001301 | 5.62E-10 |
| Smoking Initiation | rs75674569 | A | G | 0.0997 | -0.0125 | 0.00268 | 2.58E-09 |
| Smoking Initiation | rs12878369 | A | C | 0.414762 | 0.00836 | 0.00124 | 1.60E-11 |
| Smoking Initiation | rs9323328 | G | A | 0.537 | -0.00818 | 0.00162 | 2.55E-08 |
| Smoking Initiation | rs1811739 | A | G | 0.248 | 0.0112 | 0.00186 | 5.97E-10 |
| Smoking Initiation | rs8005334 | G | T | 0.36 | 0.0104 | 0.00168 | 3.44E-10 |
| Smoking Initiation | rs34940743 | G | A | 0.346 | 0.00773 | 0.0013 | 2.80E-09 |
| Smoking Initiation | rs2925128 | T | C | 0.385191 | 0.00805 | 0.00128 | 3.67E-10 |
| Smoking Initiation | rs1381287 | T | C | 0.467 | 0.00984 | 0.00163 | 1.81E-12 |
| Smoking Initiation | rs55913542 | T | G | 0.175 | 0.011 | 0.00211 | 3.25E-08 |
| Smoking Initiation | rs1435672 | C | T | 0.56 | 0.00784 | 0.00161 | 3.82E-08 |
| Smoking Initiation | rs281296 | A | G | 0.357 | 0.0143 | 0.00168 | 1.59E-20 |
| Smoking Initiation | rs1435741 | A | G | 0.432951 | 0.00904 | 0.00127 | 1.09E-12 |
| Smoking Initiation | rs56902655 | G | T | 0.136 | -0.0113 | 0.00235 | 4.09E-09 |
| Smoking Initiation | rs2289791 | T | G | 0.247 | -0.00884 | 0.00189 | 2.01E-09 |
| Smoking Initiation | rs60833441 | G | A | 0.461 | -0.00683 | 0.0016 | 2.28E-08 |
| Smoking Initiation | rs62007780 | T | G | 0.416 | -0.00787 | 0.00163 | 7.48E-10 |
| Smoking Initiation | rs4310804 | G | C | 0.247 | -0.0104 | 0.00187 | 7.55E-10 |
| Smoking Initiation | rs8027457 | C | T | 0.511 | 0.00869 | 0.0016 | 1.88E-09 |
| Smoking Initiation | rs7192140 | C | T | 0.498 | -0.00906 | 0.0016 | 3.40E-11 |
| Smoking Initiation | rs9922607 | T | C | 0.2 | -0.0122 | 0.00207 | 3.42E-12 |
| Smoking Initiation | rs9941217 | G | C | 0.352204 | -0.00892 | 0.00128 | 3.50E-12 |
| Smoking Initiation | rs7188873 | G | A | 0.612998 | 0.00972 | 0.001253 | 8.46E-15 |
| Smoking Initiation | rs6497840 | A | G | 0.707 | 0.0103 | 0.00178 | 2.01E-15 |
| Smoking Initiation | rs4785187 | A | G | 0.223 | 0.0106 | 0.00194 | 6.55E-11 |
| Smoking Initiation | rs8050598 | T | C | 0.254117 | 0.00941 | 0.00147 | 1.76E-10 |
| Smoking Initiation | rs12918191 | G | A | 0.243 | -0.0108 | 0.00188 | 3.14E-11 |
| Smoking Initiation | rs11076962 | C | T | 0.279 | 0.0101 | 0.0018 | 1.20E-10 |
| Smoking Initiation | rs9302604 | G | A | 0.435 | 0.0109 | 0.00163 | 3.29E-13 |
| Smoking Initiation | rs1139897 | A | G | 0.23 | -0.0123 | 0.00188 | 1.77E-15 |
| Smoking Initiation | rs9936784 | G | T | 0.53418 | 0.00682 | 0.00125 | 4.33E-08 |
| Smoking Initiation | rs62052916 | T | A | 0.0701 | -0.0147 | 0.00297 | 1.62E-10 |
| Smoking Initiation | rs4788676 | C | T | 0.228528 | -0.00852 | 0.00146 | 4.92E-09 |
| Smoking Initiation | rs61537885 | C | T | 0.037215 | -0.0199 | 0.00344 | 8.06E-09 |
| Smoking Initiation | rs117657830 | G | A | 0.0417 | -0.0193 | 0.00421 | 3.18E-09 |
| Smoking Initiation | rs1050847 | T | C | 0.559 | -0.0092 | 0.00165 | 7.37E-09 |
| Smoking Initiation | rs11642231 | A | G | 0.369 | -0.00864 | 0.00166 | 3.44E-09 |
| Smoking Initiation | rs11651955 | A | G | 0.499 | -0.00802 | 0.00161 | 3.74E-08 |
| Smoking Initiation | rs4790874 | T | C | 0.532 | 0.01 | 0.00163 | 8.43E-12 |
| Smoking Initiation | rs67777803 | T | G | 0.172 | -0.0136 | 0.00217 | 3.18E-13 |
| Smoking Initiation | rs2344976 | C | T | 0.612 | -0.0083 | 0.00164 | 7.98E-09 |
| Smoking Initiation | rs3764351 | A | G | 0.657 | -0.00842 | 0.0017 | 3.89E-08 |
| Smoking Initiation | rs17692129 | T | C | 0.331 | 0.0112 | 0.00185 | 4.57E-13 |
| Smoking Initiation | rs75919030 | C | T | 0.267 | -0.0114 | 0.0018 | 3.35E-13 |
| Smoking Initiation | rs2938134 | A | C | 0.673 | -0.00858 | 0.00136 | 3.14E-10 |
| Smoking Initiation | rs2587507 | C | T | 0.502 | -0.00799 | 0.00159 | 8.69E-09 |
| Smoking Initiation | rs11078713 | G | A | 0.419348 | -0.00714 | 0.00126 | 1.59E-08 |
| Smoking Initiation | rs28441558 | C | T | 0.0563 | -0.0175 | 0.00332 | 1.24E-10 |
| Smoking Initiation | rs4476253 | A | G | 0.24 | -0.0105 | 0.00188 | 5.78E-10 |
| Smoking Initiation | rs7505855 | T | C | 0.586 | -0.0103 | 0.00163 | 5.31E-11 |
| Smoking Initiation | rs8096225 | C | A | 0.703 | 0.0084 | 0.00175 | 2.63E-08 |
| Smoking Initiation | rs67050670 | G | A | 0.229 | -0.0107 | 0.00193 | 2.34E-11 |
| Smoking Initiation | rs2359180 | G | A | 0.369 | -0.00715 | 0.00206 | 4.98E-08 |
| Smoking Initiation | rs72898831 | G | A | 0.155 | -0.0151 | 0.00224 | 4.14E-12 |
| Smoking Initiation | rs8083764 | T | G | 0.306171 | -0.00778 | 0.00135 | 7.97E-09 |
| Smoking Initiation | rs1373178 | G | T | 0.588 | -0.011 | 0.00165 | 4.16E-15 |
| Smoking Initiation | rs62098013 | A | G | 0.365322 | 0.0087 | 0.0013 | 2.24E-11 |
| Smoking Initiation | rs72938304 | A | G | 0.113 | -0.0131 | 0.0026 | 1.36E-11 |
| Smoking Initiation | rs34342129 | C | T | 0.509 | -0.00727 | 0.00159 | 2.13E-08 |
| Smoking Initiation | rs11872397 | A | G | 0.253 | -0.0116 | 0.00187 | 5.20E-09 |
| Smoking Initiation | rs71367544 | T | C | 0.203 | 0.0104 | 0.00198 | 8.54E-11 |
| Smoking Initiation | rs113230003 | A | G | 0.255 | -0.0106 | 0.00189 | 1.05E-10 |
| Smoking Initiation | rs8103660 | C | T | 0.354431 | 0.00764 | 0.00129 | 3.03E-09 |
| Smoking Initiation | rs76608582 | A | C | 0.0489 | -0.027 | 0.00437 | 4.88E-09 |
| Smoking Initiation | rs10853981 | A | G | 0.33036 | 0.00713 | 0.00131 | 4.88E-08 |
| Smoking Initiation | rs117734003 | C | G | 0.0673 | 0.015 | 0.00338 | 2.57E-09 |
| Smoking Initiation | rs1126757 | T | C | 0.473 | 0.0077 | 0.00161 | 2.92E-08 |
| Smoking Initiation | rs13392222 | C | A | 0.139 | -0.0121 | 0.0023 | 1.93E-10 |
| Smoking Initiation | rs1901477 | G | A | 0.511 | 0.0164 | 0.0016 | 2.07E-31 |
| Smoking Initiation | rs11889814 | C | A | 0.128 | -0.0101 | 0.00184 | 3.44E-08 |
| Smoking Initiation | rs3811038 | C | T | 0.279 | 0.0102 | 0.00179 | 1.58E-11 |
| Smoking Initiation | rs75210106 | T | C | 0.176676 | -0.00918 | 0.00164 | 2.33E-08 |
| Smoking Initiation | rs34399632 | G | A | 0.232 | 0.0101 | 0.00185 | 1.46E-10 |
| Smoking Initiation | rs74697736 | A | G | 0.287239 | 0.0107 | 0.001357 | 2.43E-15 |
| Smoking Initiation | rs6756212 | T | C | 0.535 | -0.0188 | 0.0016 | 3.49E-40 |
| Smoking Initiation | rs16826827 | C | T | 0.124 | -0.0128 | 0.00243 | 9.17E-09 |
| Smoking Initiation | rs1445649 | C | T | 0.538 | 0.011 | 0.0016 | 8.48E-16 |
| Smoking Initiation | rs1722666 | T | C | 0.732 | 0.00771 | 0.001378 | 2.17E-08 |
| Smoking Initiation | rs11678980 | A | G | 0.45 | 0.00893 | 0.001295 | 5.19E-12 |
| Smoking Initiation | rs12474587 | T | G | 0.429 | 0.0129 | 0.00162 | 4.83E-21 |
| Smoking Initiation | rs357304 | C | T | 0.727 | 0.00933 | 0.00183 | 5.40E-09 |
| Smoking Initiation | rs13007361 | A | G | 0.208 | 0.0107 | 0.00201 | 2.29E-08 |
| Smoking Initiation | rs7600835 | A | G | 0.342 | -0.00868 | 0.00173 | 1.80E-08 |
| Smoking Initiation | rs6750529 | T | C | 0.744 | 0.0114 | 0.00184 | 9.26E-12 |
| Smoking Initiation | rs17229285 | T | C | 0.505 | -0.0103 | 0.0016 | 1.27E-09 |
| Smoking Initiation | rs3115418 | C | T | 0.454 | -0.00876 | 0.0016 | 2.79E-08 |
| Smoking Initiation | rs62193862 | A | G | 0.0999 | 0.0149 | 0.00279 | 1.99E-08 |
| Smoking Initiation | rs1022376 | C | T | 0.515821 | -0.0071 | 0.001258 | 1.66E-08 |
| Smoking Initiation | rs4674916 | A | C | 0.327671 | -0.00861 | 0.0013 | 3.06E-11 |
| Smoking Initiation | rs61533748 | C | T | 0.384 | 0.00915 | 0.00166 | 2.82E-11 |
| Smoking Initiation | rs4674993 | G | A | 0.2 | -0.0133 | 0.002 | 4.85E-14 |
| Smoking Initiation | rs114976176 | C | A | 0.351571 | -0.00746 | 0.00128 | 6.04E-09 |
| Smoking Initiation | rs72790288 | A | G | 0.0282 | -0.0225 | 0.00483 | 3.28E-09 |
| Smoking Initiation | rs2710634 | C | T | 0.521 | -0.00942 | 0.00162 | 3.36E-12 |
| Smoking Initiation | rs62106258 | C | T | 0.047329 | -0.0242 | 0.003195 | 3.33E-14 |
| Smoking Initiation | rs62137126 | G | A | 0.121094 | -0.0114 | 0.00188 | 1.31E-09 |
| Smoking Initiation | rs1004787 | A | G | 0.552 | 0.0152 | 0.00162 | 1.11E-28 |
| Smoking Initiation | rs7598402 | G | C | 0.492084 | -0.00709 | 0.00123 | 7.38E-09 |
| Smoking Initiation | rs10490159 | T | C | 0.394 | 0.0119 | 0.00164 | 3.86E-11 |
| Smoking Initiation | rs1518393 | C | A | 0.619 | 0.00988 | 0.00168 | 1.30E-10 |
| Smoking Initiation | rs17616642 | G | A | 0.24687 | -0.00797 | 0.00142 | 2.10E-08 |
| Smoking Initiation | rs6730325 | A | G | 0.609782 | -0.00701 | 0.00125 | 2.10E-08 |
| Smoking Initiation | rs2539706 | A | G | 0.529947 | 0.00789 | 0.00124 | 1.95E-10 |
| Smoking Initiation | rs7585579 | G | C | 0.499 | 0.00998 | 0.00163 | 5.48E-15 |
| Smoking Initiation | rs1863161 | A | G | 0.56094 | 0.00746 | 0.00125 | 2.34E-09 |
| Smoking Initiation | rs359247 | T | A | 0.638652 | 0.0105 | 0.00127 | 9.89E-17 |
| Smoking Initiation | rs6731872 | G | T | 0.826 | 0.0182 | 0.00213 | 5.35E-21 |
| Smoking Initiation | rs62180324 | A | G | 0.212 | -0.0103 | 0.00193 | 3.91E-10 |
| Smoking Initiation | rs6750107 | A | G | 0.386875 | 0.00705 | 0.00127 | 2.60E-08 |
| Smoking Initiation | rs12714017 | C | T | 0.511 | 0.00877 | 0.00162 | 3.65E-09 |
| Smoking Initiation | rs56208390 | G | A | 0.123 | 0.011 | 0.00242 | 2.68E-08 |
| Smoking Initiation | rs11692435 | A | G | 0.0848 | 0.027734 | 0.071217 | 4.47E-08 |
| Smoking Initiation | rs6050446 | G | A | 0.971 | 0.0351 | 0.00488 | 8.80E-13 |
| Smoking Initiation | rs6058782 | T | C | 0.908 | 0.0144 | 0.00214 | 1.78E-11 |
| Smoking Initiation | rs1555445 | T | A | 0.318 | 0.0103 | 0.00174 | 7.75E-12 |
| Smoking Initiation | rs6073075 | A | T | 0.824 | -0.0108 | 0.00215 | 2.44E-08 |
| Smoking Initiation | rs910912 | C | T | 0.739 | -0.0112 | 0.00183 | 7.82E-09 |
| Smoking Initiation | rs6011779 | T | C | 0.806 | -0.0104 | 0.00206 | 2.83E-09 |
| Smoking Initiation | rs3810496 | C | T | 0.619436 | 0.00785 | 0.0013 | 1.54E-09 |
| Smoking Initiation | rs4818005 | A | G | 0.581 | -0.0106 | 0.00164 | 1.09E-14 |
| Smoking Initiation | rs139896 | C | T | 0.648 | 0.00891 | 0.00168 | 7.14E-09 |
| Smoking Initiation | rs4822102 | T | C | 0.618 | -0.0083 | 0.00163 | 2.78E-10 |
| Smoking Initiation | rs9627272 | C | G | 0.407 | -0.0103 | 0.00175 | 2.42E-09 |
| Smoking Initiation | rs6437769 | T | C | 0.581 | 0.00841 | 0.00161 | 3.74E-08 |
| Smoking Initiation | rs9288999 | A | G | 0.735 | 0.0105 | 0.00183 | 1.50E-09 |
| Smoking Initiation | rs6438436 | T | C | 0.816 | 0.0151 | 0.00209 | 5.33E-14 |
| Smoking Initiation | rs12053870 | G | T | 0.541511 | 0.00754 | 0.00123 | 1.02E-09 |
| Smoking Initiation | rs9826984 | A | G | 0.542 | -0.0085 | 0.00161 | 3.87E-08 |
| Smoking Initiation | rs2279829 | T | C | 0.216 | -0.0087 | 0.00194 | 2.05E-08 |
| Smoking Initiation | rs2319545 | A | C | 0.149099 | 0.0112 | 0.00173 | 8.30E-11 |
| Smoking Initiation | rs10935779 | T | C | 0.415 | -0.00777 | 0.00162 | 2.95E-08 |
| Smoking Initiation | rs963354 | A | C | 0.687 | 0.00733 | 0.001337 | 4.21E-08 |
| Smoking Initiation | rs1714521 | C | A | 0.411 | -0.00978 | 0.00164 | 3.07E-10 |
| Smoking Initiation | rs1449012 | T | C | 0.463 | -0.00747 | 0.00124 | 1.77E-09 |
| Smoking Initiation | rs9850597 | A | G | 0.816 | -0.0103 | 0.00207 | 1.65E-08 |
| Smoking Initiation | rs748832 | G | A | 0.371 | 0.00931 | 0.00165 | 6.60E-11 |
| Smoking Initiation | rs1187820 | T | C | 0.439 | -0.00706 | 0.00127 | 2.69E-08 |
| Smoking Initiation | rs16828799 | T | G | 0.156 | 0.00986 | 0.00215 | 1.83E-08 |
| Smoking Initiation | rs9841807 | T | C | 0.273 | 0.00919 | 0.00179 | 1.35E-08 |
| Smoking Initiation | rs7631379 | C | T | 0.206 | 0.0117 | 0.00206 | 3.94E-11 |
| Smoking Initiation | rs11713899 | C | A | 0.171 | 0.0108 | 0.00215 | 3.15E-08 |
| Smoking Initiation | rs10446419 | G | A | 0.207 | -0.011 | 0.00208 | 5.05E-10 |
| Smoking Initiation | rs13319205 | A | T | 0.29 | 0.00804 | 0.00136 | 3.77E-09 |
| Smoking Initiation | rs3172494 | T | G | 0.115 | -0.014 | 0.00193 | 3.40E-13 |
| Smoking Initiation | rs2526390 | T | C | 0.334 | 0.0116 | 0.00171 | 3.62E-14 |
| Smoking Initiation | rs2276825 | C | T | 0.245 | 0.0112 | 0.00188 | 1.89E-10 |
| Smoking Initiation | rs2306866 | T | A | 0.614 | -0.00801 | 0.001257 | 1.89E-10 |
| Smoking Initiation | rs73831818 | G | A | 0.057 | 0.0172 | 0.00337 | 5.46E-09 |
| Smoking Initiation | rs1910236 | A | G | 0.469 | 0.0083 | 0.00163 | 9.91E-09 |
| Smoking Initiation | rs7640107 | T | C | 0.430789 | -0.00679 | 0.00123 | 3.46E-08 |
| Smoking Initiation | rs2734390 | G | A | 0.372 | 0.00774 | 0.00167 | 2.09E-08 |
| Smoking Initiation | rs221988 | C | A | 0.384 | -0.00787 | 0.00172 | 1.43E-08 |
| Smoking Initiation | rs2196356 | C | G | 0.288886 | -0.00923 | 0.00138 | 2.45E-11 |
| Smoking Initiation | rs11128203 | A | T | 0.53 | 0.0109 | 0.00165 | 1.29E-15 |
| Smoking Initiation | rs62246017 | A | G | 0.322639 | -0.00791 | 0.00133 | 3.03E-09 |
| Smoking Initiation | rs4543050 | T | A | 0.816 | 0.0138 | 0.0021 | 1.45E-11 |
| Smoking Initiation | rs6782116 | T | C | 0.415 | -0.00713 | 0.00167 | 1.46E-08 |
| Smoking Initiation | rs13066050 | T | C | 0.208 | 0.00947 | 0.00195 | 1.93E-09 |
| Smoking Initiation | rs12633090 | C | G | 0.182 | -0.0111 | 0.0016 | 3.16E-12 |
| Smoking Initiation | rs1549979 | T | C | 0.615 | -0.0133 | 0.00165 | 8.80E-21 |
| Smoking Initiation | rs57153235 | G | T | 0.318 | -0.00949 | 0.001342 | 1.56E-12 |
| Smoking Initiation | rs3934797 | A | G | 0.182 | -0.0128 | 0.00212 | 1.12E-10 |
| Smoking Initiation | rs71602617 | T | C | 0.216 | -0.0098 | 0.00195 | 2.10E-08 |
| Smoking Initiation | rs7696257 | A | G | 0.366 | 0.00737 | 0.00168 | 6.78E-09 |
| Smoking Initiation | rs13109980 | A | G | 0.326 | -0.012 | 0.0017 | 3.37E-16 |
| Smoking Initiation | rs1116690 | G | A | 0.742 | 0.00963 | 0.00186 | 2.16E-08 |
| Smoking Initiation | rs13110073 | C | T | 0.395 | -0.0132 | 0.00164 | 3.24E-21 |
| Smoking Initiation | rs28717373 | T | C | 0.356165 | -0.00827 | 0.001337 | 6.16E-10 |
| Smoking Initiation | rs4140932 | A | T | 0.431 | -0.00727 | 0.00162 | 4.89E-08 |
| Smoking Initiation | rs62340589 | C | G | 0.201 | 0.0102 | 0.00198 | 4.31E-08 |
| Smoking Initiation | rs12642744 | T | G | 0.744 | -0.00807 | 0.001454 | 2.82E-08 |
| Smoking Initiation | rs59537158 | T | C | 0.214 | 0.0127 | 0.00202 | 4.62E-13 |
| Smoking Initiation | rs1389171 | A | T | 0.241 | -0.00843 | 0.001437 | 4.45E-09 |
| Smoking Initiation | rs55944129 | C | T | 0.267 | -0.00841 | 0.00138 | 1.06E-09 |
| Smoking Initiation | rs58400863 | A | G | 0.347 | -0.0102 | 0.00171 | 4.89E-14 |
| Smoking Initiation | rs7657022 | G | A | 0.489 | 0.00914 | 0.0016 | 7.34E-13 |
| Smoking Initiation | rs112725451 | T | C | 0.169 | 0.014 | 0.00215 | 1.65E-14 |
| Smoking Initiation | rs1160685 | G | C | 0.45 | 0.00992 | 0.00162 | 2.31E-09 |
| Smoking Initiation | rs1435479 | T | G | 0.28748 | 0.00784 | 0.001346 | 5.68E-09 |
| Smoking Initiation | rs72780746 | C | T | 0.173 | -0.014 | 0.00211 | 2.05E-14 |
| Smoking Initiation | rs10060196 | A | C | 0.580606 | 0.00879 | 0.00124 | 1.29E-12 |
| Smoking Initiation | rs72789626 | A | T | 0.136 | -0.0157 | 0.00229 | 5.13E-12 |
| Smoking Initiation | rs17165769 | G | A | 0.394872 | 0.00762 | 0.001246 | 9.56E-10 |
| Smoking Initiation | rs329124 | G | A | 0.428 | -0.00865 | 0.00162 | 1.96E-10 |
| Smoking Initiation | rs1385108 | T | C | 0.239 | 0.0123 | 0.00188 | 3.84E-10 |
| Smoking Initiation | rs1173461 | T | C | 0.327 | 0.0083 | 0.00172 | 9.51E-10 |
| Smoking Initiation | rs11956866 | G | T | 0.567 | -0.00813 | 0.00161 | 7.82E-09 |
| Smoking Initiation | rs3909281 | G | T | 0.536 | 0.0101 | 0.001228 | 1.62E-16 |
| Smoking Initiation | rs3843905 | T | C | 0.403 | -0.00726 | 0.001245 | 5.41E-09 |
| Smoking Initiation | rs79476395 | G | A | 0.0726 | 0.0162 | 0.00238 | 1.04E-11 |
| Smoking Initiation | rs6890961 | T | C | 0.624 | -0.00929 | 0.001266 | 2.13E-13 |
| Smoking Initiation | rs4044321 | G | A | 0.644 | -0.0139 | 0.00168 | 1.75E-17 |
| Smoking Initiation | rs2173019 | A | T | 0.177 | 0.0135 | 0.001601 | 2.98E-17 |
| Smoking Initiation | rs10042827 | C | T | 0.681 | 0.00947 | 0.00173 | 9.41E-10 |
| Smoking Initiation | rs359431 | T | C | 0.56 | -0.00762 | 0.0016 | 3.16E-08 |
| Smoking Initiation | rs12517438 | G | T | 0.538 | 0.0109 | 0.00161 | 1.89E-09 |
| Smoking Initiation | rs35375873 | C | G | 0.11 | -0.0162 | 0.00268 | 3.29E-11 |
| Smoking Initiation | rs986714 | T | A | 0.445 | -0.0077 | 0.00161 | 4.13E-10 |
| Smoking Initiation | rs71592686 | C | T | 0.274 | 0.0121 | 0.00179 | 3.85E-13 |
| Smoking Initiation | rs2028269 | A | G | 0.399 | 0.00773 | 0.00124 | 5.19E-10 |
| Smoking Initiation | rs6874731 | G | T | 0.484 | 0.00899 | 0.00161 | 1.83E-09 |
| Smoking Initiation | rs6452785 | T | C | 0.474 | -0.0152 | 0.00161 | 4.69E-26 |
| Smoking Initiation | rs10805858 | T | A | 0.335286 | 0.00874 | 0.001301 | 1.88E-11 |
| Smoking Initiation | rs42417 | T | C | 0.691 | 0.0116 | 0.00174 | 8.27E-10 |
| Smoking Initiation | rs6936160 | T | C | 0.698 | 0.00963 | 0.00133 | 4.20E-13 |
| Smoking Initiation | rs12530388 | C | A | 0.511 | -0.0104 | 0.0016 | 5.83E-13 |
| Smoking Initiation | rs3800227 | G | A | 0.742 | 0.0104 | 0.00184 | 3.64E-09 |
| Smoking Initiation | rs118202 | T | G | 0.812 | -0.0196 | 0.00204 | 1.90E-29 |
| Smoking Initiation | rs73008357 | C | A | 0.121 | -0.0121 | 0.00244 | 2.44E-08 |
| Smoking Initiation | rs9331343 | C | T | 0.568 | -0.00875 | 0.00173 | 3.90E-08 |
| Smoking Initiation | rs10698713 | A | G | 0.0544 | -0.0184 | 0.00368 | 2.38E-09 |
| Smoking Initiation | rs1737329 | G | C | 0.742 | 0.00935 | 0.00182 | 5.08E-09 |
| Smoking Initiation | rs1059490 | C | T | 0.367 | -0.0102 | 0.0017 | 2.16E-12 |
| Smoking Initiation | rs6932350 | A | T | 0.454655 | 0.00725 | 0.00124 | 5.13E-09 |
| Smoking Initiation | rs1150668 | G | T | 0.419 | -0.01 | 0.00173 | 8.54E-13 |
| Smoking Initiation | rs1632941 | C | T | 0.46 | -0.00767 | 0.001242 | 6.67E-10 |
| Smoking Initiation | rs3218116 | T | C | 0.256 | -0.0105 | 0.00182 | 1.05E-11 |
| Smoking Initiation | rs160631 | G | T | 0.731 | -0.00981 | 0.0018 | 1.87E-09 |
| Smoking Initiation | rs7743165 | G | T | 0.495 | 0.0102 | 0.0016 | 4.15E-14 |
| Smoking Initiation | rs10945141 | A | G | 0.263 | 0.00936 | 0.00181 | 3.59E-10 |
| Smoking Initiation | rs17554906 | C | G | 0.444 | 0.00806 | 0.00161 | 3.14E-08 |
| Smoking Initiation | rs619087 | G | A | 0.422 | 0.00809 | 0.00162 | 3.10E-08 |
| Smoking Initiation | rs6568832 | A | G | 0.753851 | 0.00903 | 0.001415 | 1.74E-10 |
| Smoking Initiation | rs12195240 | A | G | 0.285 | 0.013 | 0.00178 | 1.08E-18 |
| Smoking Initiation | rs11766326 | C | T | 0.506 | -0.00821 | 0.00161 | 1.79E-11 |
| Smoking Initiation | rs6968380 | A | G | 0.681 | -0.013 | 0.00174 | 1.05E-17 |
| Smoking Initiation | rs10233018 | G | A | 0.516 | 0.0149 | 0.00161 | 4.77E-22 |
| Smoking Initiation | rs10953957 | A | G | 0.386 | 0.00907 | 0.00167 | 3.66E-08 |
| Smoking Initiation | rs77283305 | A | G | 0.305819 | -0.00728 | 0.00133 | 3.91E-08 |
| Smoking Initiation | rs10279261 | A | G | 0.618 | -0.0114 | 0.00169 | 6.05E-13 |
| Smoking Initiation | rs1561112 | C | T | 0.412815 | -0.00742 | 0.00126 | 3.84E-09 |
| Smoking Initiation | rs10272990 | C | T | 0.327622 | -0.0102 | 0.00132 | 1.27E-14 |
| Smoking Initiation | rs6948707 | G | T | 0.419 | 0.0129 | 0.00163 | 4.24E-21 |
| Smoking Initiation | rs10259715 | A | T | 0.209918 | -0.00974 | 0.00168 | 6.42E-09 |
| Smoking Initiation | rs13237637 | C | G | 0.485 | -0.0123 | 0.0016 | 1.54E-20 |
| Smoking Initiation | rs7809303 | A | G | 0.325 | -0.0115 | 0.0017 | 3.48E-15 |
| Smoking Initiation | rs7802996 | T | C | 0.166 | -0.0123 | 0.00211 | 1.06E-09 |
| Smoking Initiation | rs1030015 | T | G | 0.519564 | 0.00682 | 0.001218 | 2.15E-08 |
| Smoking Initiation | rs4727189 | C | T | 0.344 | 0.00772 | 0.00168 | 3.00E-08 |
| Smoking Initiation | rs76841737 | G | C | 0.103 | -0.0118 | 0.00261 | 3.26E-08 |
| Smoking Initiation | rs11768481 | A | C | 0.34 | -0.0111 | 0.00176 | 5.23E-12 |
| Smoking Initiation | rs1799068 | T | G | 0.379 | 0.00838 | 0.00164 | 2.59E-10 |
| Smoking Initiation | rs13437771 | G | A | 0.155 | -0.015 | 0.00221 | 1.39E-14 |
| Smoking Initiation | rs2952251 | G | A | 0.74437 | 0.00816 | 0.00149 | 4.24E-08 |
| Smoking Initiation | rs4326350 | G | C | 0.493 | -0.00894 | 0.00162 | 5.16E-12 |
| Smoking Initiation | rs290601 | T | C | 0.274 | 0.0085 | 0.00178 | 1.14E-08 |
| Smoking Initiation | rs11783093 | T | C | 0.158 | -0.0253 | 0.00225 | 2.07E-41 |
| Smoking Initiation | rs7836565 | T | C | 0.718 | -0.00923 | 0.00178 | 4.36E-08 |
| Smoking Initiation | rs13261666 | T | G | 0.517 | -0.0117 | 0.0016 | 4.36E-15 |
| Smoking Initiation | rs3850736 | G | C | 0.474 | 0.0109 | 0.00161 | 6.43E-14 |
| Smoking Initiation | rs2063976 | T | C | 0.664955 | -0.00968 | 0.001294 | 7.45E-14 |
| Smoking Initiation | rs6993429 | A | C | 0.453 | -0.00984 | 0.00161 | 9.87E-14 |
| Smoking Initiation | rs6986430 | C | T | 0.222377 | -0.0117 | 0.00147 | 1.99E-15 |
| Smoking Initiation | rs9987376 | G | T | 0.574251 | -0.00982 | 0.001237 | 2.01E-15 |
| Smoking Initiation | rs6474609 | A | T | 0.586731 | -0.00757 | 0.001257 | 1.71E-09 |
| Smoking Initiation | rs1931431 | C | G | 0.478 | 0.00884 | 0.0016 | 8.56E-13 |
| Smoking Initiation | rs1927901 | C | T | 0.553 | -0.00859 | 0.00161 | 3.10E-08 |
| Smoking Initiation | rs4837631 | T | C | 0.446 | -0.00878 | 0.0016 | 2.03E-09 |
| Smoking Initiation | rs1759433 | A | G | 0.48 | 0.0076 | 0.0016 | 1.69E-09 |
| Smoking Initiation | rs34553878 | G | A | 0.111 | 0.027377 | 0.067088 | 1.17E-09 |
| Smoking Initiation | rs7026534 | G | T | 0.703821 | -0.00793 | 0.00133 | 2.68E-09 |
| Smoking Initiation | rs10858334 | G | C | 0.14 | 0.013 | 0.0024 | 1.18E-09 |
| Smoking Initiation | rs7867822 | G | A | 0.673 | -0.00839 | 0.0017 | 2.76E-08 |
| Smoking Initiation | rs10966092 | C | T | 0.267 | -0.0104 | 0.00181 | 1.12E-12 |
| Smoking Initiation | rs10969352 | A | T | 0.5 | 0.00722 | 0.0016 | 1.82E-08 |
| Smoking Initiation | rs3847244 | T | C | 0.47 | 0.01 | 0.00165 | 2.60E-13 |
| Smoking Initiation | rs11791671 | T | C | 0.067315 | 0.0134 | 0.00244 | 4.24E-08 |
| Smoking Initiation | rs4877285 | A | G | 0.668249 | -0.00881 | 0.00131 | 2.10E-11 |
| Smoking Initiation | rs1930371 | T | C | 0.241 | -0.0094 | 0.00189 | 7.09E-09 |
| Smoking Initiation | rs7024924 | C | T | 0.174 | 0.0108 | 0.00213 | 1.90E-08 |
| Smoking Initiation | rs2378662 | A | G | 0.541 | 0.00879 | 0.00162 | 2.67E-09 |
| coffee | rs2472297 | T | C | 0.2641 | 0.0447 | 0.0017 | 9.39E-157 |
| coffee | rs4410790 | C | T | 0.633 | 0.0387 | 0.0015 | 4.48E-142 |
| coffee | rs1057868 | T | C | 0.2851 | 0.0197 | 0.0016 | 1.12E-33 |
| coffee | rs73073176 | C | T | 0.8701 | 0.023 | 0.0022 | 1.54E-25 |
| coffee | rs9937053 | A | G | 0.424 | 0.0142 | 0.0015 | 1.63E-21 |
| coffee | rs1260326 | C | T | 0.6047 | 0.0136 | 0.0015 | 1.41E-19 |
| coffee | rs66723169 | A | C | 0.232 | 0.0155 | 0.0018 | 8.41E-19 |
| coffee | rs34060476 | G | A | 0.1337 | 0.019 | 0.0022 | 1.61E-18 |
| coffee | rs12699844 | C | T | 0.468 | 0.0122 | 0.0015 | 1.84E-16 |
| coffee | rs10865548 | G | A | 0.8276 | 0.016 | 0.0019 | 2.20E-16 |
| coffee | rs56113850 | C | T | 0.5776 | 0.0121 | 0.0015 | 8.02E-16 |
| coffee | rs2330783 | G | T | 0.986 | 0.0442 | 0.0063 | 1.71E-12 |
| coffee | rs10127720 | C | T | 0.7403 | 0.0116 | 0.0017 | 6.56E-12 |
| coffee | rs16903275 | A | C | 0.1573 | 0.0138 | 0.002 | 7.45E-12 |
| coffee | rs6062682 | T | C | 0.4641 | 0.01 | 0.0015 | 2.15E-11 |
| coffee | rs2465037 | C | A | 0.656 | 0.0103 | 0.0016 | 3.54E-11 |
| coffee | rs2199936 | G | A | 0.8866 | 0.0153 | 0.0023 | 3.93E-11 |
| coffee | rs597045 | A | T | 0.6945 | 0.0106 | 0.0016 | 5.95E-11 |
| coffee | rs57918684 | A | G | 0.1551 | 0.0129 | 0.002 | 2.41E-10 |
| coffee | rs117692895 | C | G | 0.0129 | 0.0421 | 0.0067 | 3.72E-10 |
| coffee | rs574367 | T | G | 0.2104 | 0.0112 | 0.0018 | 5.01E-10 |
| coffee | rs586346 | T | C | 0.3648 | 0.0095 | 0.0015 | 6.73E-10 |
| coffee | rs993885 | G | A | 0.6326 | 0.0092 | 0.0015 | 1.92E-09 |
| coffee | rs2597979 | G | C | 0.1964 | 0.0111 | 0.0019 | 2.70E-09 |
| coffee | rs2297508 | G | C | 0.6489 | 0.0092 | 0.0015 | 3.27E-09 |
| coffee | rs62104180 | G | A | 0.95 | 0.0199 | 0.0034 | 4.86E-09 |
| coffee | rs3810291 | A | G | 0.6772 | 0.0092 | 0.0016 | 6.55E-09 |
| coffee | rs6124969 | T | C | 0.3642 | 0.0088 | 0.0015 | 8.84E-09 |
| coffee | rs75347775 | A | G | 0.2454 | 0.0097 | 0.0017 | 1.44E-08 |
| coffee | rs2613458 | C | A | 0.719 | 0.0093 | 0.0016 | 1.46E-08 |
| coffee | rs1956218 | G | A | 0.5587 | 0.0084 | 0.0015 | 1.86E-08 |
| coffee | rs4719497 | T | C | 0.8652 | 0.0121 | 0.0022 | 2.13E-08 |
| coffee | rs10997940 | C | T | 0.5979 | 0.0083 | 0.0015 | 2.97E-08 |
| coffee | rs17687539 | A | G | 0.792 | 0.01 | 0.0018 | 3.91E-08 |
| coffee | rs8056750 | T | C | 0.3595 | 0.0087 | 0.0016 | 4.10E-08 |
| coffee | rs2521501 | A | T | 0.6768 | 0.0087 | 0.0016 | 4.14E-08 |
| coffee | rs13054099 | T | C | 0.7399 | 0.0091 | 0.0017 | 5.09E-08 |
| coffee | rs4475789 | G | T | 0.6547 | 0.0083 | 0.0016 | 9.60E-08 |
| tea | rs2472297 | T | C | 0.2614 | 0.0247 | 0.0017 | 1.18E-45 |
| tea | rs4410790 | C | T | 0.6316 | 0.018 | 0.0016 | 3.48E-30 |
| tea | rs9624470 | A | G | 0.5794 | 0.0126 | 0.0015 | 3.96E-16 |
| tea | rs2504716 | T | C | 0.2098 | 0.0134 | 0.0019 | 5.49E-13 |
| tea | rs4817505 | C | T | 0.3882 | 0.0109 | 0.0016 | 2.43E-12 |
| tea | rs319277 | A | G | 0.8366 | 0.0144 | 0.0021 | 2.52E-12 |
| tea | rs73169830 | C | T | 0.0764 | 0.0192 | 0.0029 | 2.45E-11 |
| tea | rs713598 | G | C | 0.401 | 0.0098 | 0.0016 | 2.72E-10 |
| tea | rs6872796 | A | C | 0.715 | 0.0105 | 0.0017 | 7.71E-10 |
| tea | rs2117137 | G | A | 0.4048 | 0.0094 | 0.0015 | 1.09E-09 |
| tea | rs56163935 | C | T | 0.1811 | 0.012 | 0.002 | 1.23E-09 |
| tea | rs2074551 | C | G | 0.2733 | 0.0105 | 0.0018 | 6.51E-09 |
| tea | rs56188862 | T | C | 0.6128 | 0.0088 | 0.0016 | 1.81E-08 |
| tea | rs6462899 | A | T | 0.6238 | 0.0087 | 0.0016 | 2.26E-08 |
| tea | rs349356 | T | G | 0.7046 | 0.0092 | 0.0017 | 3.40E-08 |
| tea | rs2273446 | G | C | 0.1725 | 0.0111 | 0.002 | 3.74E-08 |
| tea | rs2271961 | C | T | 0.4957 | 0.0081 | 0.0015 | 8.56E-08 |
| tea | rs2813703 | A | G | 0.5741 | 0.0082 | 0.0015 | 9.60E-08 |
| bitter nonalcoholic beverages | rs2472297 | T | C | 0.2611 | 0.0385 | 0.0018 | 1.36E-97 |
| bitter nonalcoholic beverages | rs4410790 | C | T | 0.63 | 0.0305 | 0.0017 | 2.80E-75 |
| bitter nonalcoholic beverages | rs1057868 | T | C | 0.2857 | 0.0176 | 0.0018 | 3.98E-23 |
| bitter nonalcoholic beverages | rs7791070 | T | C | 0.7663 | 0.0175 | 0.0019 | 3.94E-20 |
| bitter nonalcoholic beverages | rs56113850 | C | T | 0.5766 | 0.0107 | 0.0016 | 6.67E-11 |
| bitter nonalcoholic beverages | rs9607819 | G | C | 0.8068 | 0.0131 | 0.002 | 1.82E-10 |
| bitter nonalcoholic beverages | rs12405726 | A | G | 0.3514 | 0.0107 | 0.0017 | 2.49E-10 |
| bitter nonalcoholic beverages | rs1808056 | A | G | 0.4618 | 0.0096 | 0.0016 | 5.00E-09 |
| bitter nonalcoholic beverages | rs35855035 | T | C | 0.2166 | 0.0108 | 0.002 | 3.45E-08 |
| bitter nonalcoholic beverages | rs3788372 | A | G | 0.5926 | 0.0088 | 0.0016 | 8.18E-08 |
| total bitter beverages | rs2472297 | T | C | 0.2609 | 0.0334 | 0.0018 | 3.70E-74 |
| total bitter beverages | rs4410790 | C | T | 0.6298 | 0.0267 | 0.0017 | 5.04E-58 |
| total bitter beverages | rs17685 | A | G | 0.2785 | 0.0147 | 0.0018 | 2.69E-16 |
| total bitter beverages | rs1260326 | C | T | 0.6058 | 0.0111 | 0.0016 | 1.38E-11 |
| total bitter beverages | rs56113850 | C | T | 0.5766 | 0.0107 | 0.0016 | 8.17E-11 |
| total bitter beverages | rs5758274 | A | G | 0.6674 | 0.0103 | 0.0017 | 1.76E-09 |
| total bitter beverages | rs7693253 | A | G | 0.4426 | 0.0097 | 0.0016 | 2.15E-09 |
| total bitter beverages | rs1481012 | A | G | 0.8869 | 0.0146 | 0.0025 | 8.13E-09 |
| total bitter beverages | rs1808056 | A | G | 0.4616 | 0.0093 | 0.0016 | 1.54E-08 |
| total bitter beverages | rs1393320 | G | T | 0.4515 | 0.0086 | 0.0016 | 9.65E-08 |
| bitter alcoholic beverages | rs1229984 | C | T | 0.9725 | 0.0821 | 0.0033 | 1.57E-140 |
| bitter alcoholic beverages | rs1260326 | C | T | 0.6034 | 0.0128 | 0.001 | 2.44E-35 |
| bitter alcoholic beverages | rs11940694 | G | A | 0.6023 | 0.0125 | 0.001 | 3.88E-33 |
| bitter alcoholic beverages | rs1004787 | A | G | 0.5328 | 0.0087 | 0.001 | 1.50E-17 |
| bitter alcoholic beverages | rs62305780 | G | C | 0.1025 | 0.0144 | 0.0017 | 2.61E-17 |
| bitter alcoholic beverages | rs17028973 | T | C | 0.8118 | 0.0103 | 0.0013 | 5.36E-15 |
| bitter alcoholic beverages | rs13135092 | A | G | 0.9176 | 0.0141 | 0.0019 | 3.48E-14 |
| bitter alcoholic beverages | rs7935528 | A | G | 0.4507 | 0.0077 | 0.001 | 5.22E-14 |
| bitter alcoholic beverages | rs4279114 | G | C | 0.2471 | 0.0078 | 0.0012 | 2.42E-11 |
| bitter alcoholic beverages | rs113443718 | G | A | 0.6913 | 0.0073 | 0.0011 | 2.65E-11 |
| bitter alcoholic beverages | rs13157159 | T | C | 0.2078 | 0.0082 | 0.0013 | 7.51E-11 |
| bitter alcoholic beverages | rs1724409 | T | G | 0.7415 | 0.0075 | 0.0012 | 1.02E-10 |
| bitter alcoholic beverages | rs4726481 | G | T | 0.601 | 0.0067 | 0.001 | 1.87E-10 |
| bitter alcoholic beverages | rs1788820 | G | A | 0.6548 | 0.0067 | 0.0011 | 2.42E-10 |
| bitter alcoholic beverages | rs2622167 | A | G | 0.4267 | 0.0066 | 0.0011 | 4.34E-10 |
| bitter alcoholic beverages | rs324012 | T | C | 0.4488 | 0.0062 | 0.001 | 9.35E-10 |
| bitter alcoholic beverages | rs756747 | G | T | 0.5222 | 0.0061 | 0.001 | 2.98E-09 |
| bitter alcoholic beverages | rs11692435 | A | G | 0.084 | 0.0112 | 0.0019 | 4.26E-09 |
| bitter alcoholic beverages | rs74424378 | T | G | 0.7621 | 0.007 | 0.0012 | 5.07E-09 |
| bitter alcoholic beverages | rs9607812 | G | A | 0.8113 | 0.0075 | 0.0013 | 7.38E-09 |
| bitter alcoholic beverages | rs11648570 | C | T | 0.1074 | 0.0095 | 0.0017 | 7.47E-09 |
| bitter alcoholic beverages | rs13413953 | T | G | 0.6396 | 0.0061 | 0.0011 | 8.38E-09 |
| bitter alcoholic beverages | rs10892761 | C | T | 0.427 | 0.0059 | 0.001 | 1.04E-08 |
| bitter alcoholic beverages | rs196376 | G | A | 0.5768 | 0.0059 | 0.001 | 1.11E-08 |
| bitter alcoholic beverages | rs12945870 | T | C | 0.5683 | 0.0058 | 0.001 | 1.91E-08 |
| bitter alcoholic beverages | rs503397 | T | C | 0.2822 | 0.0064 | 0.0011 | 2.55E-08 |
| bitter alcoholic beverages | rs3803800 | G | A | 0.7888 | 0.0069 | 0.0012 | 2.72E-08 |
| bitter alcoholic beverages | rs28616142 | T | C | 0.395 | 0.0058 | 0.001 | 2.88E-08 |
| bitter alcoholic beverages | rs10894762 | T | C | 0.7723 | 0.0067 | 0.0012 | 2.93E-08 |
| bitter alcoholic beverages | rs62135521 | G | T | 0.9513 | 0.0131 | 0.0024 | 3.02E-08 |
| bitter alcoholic beverages | rs928736 | C | T | 0.3064 | 0.0061 | 0.0011 | 3.97E-08 |
| bitter alcoholic beverages | rs17520061 | C | G | 0.8584 | 0.008 | 0.0015 | 5.91E-08 |
| bitter alcoholic beverages | rs973894 | C | G | 0.2794 | 0.0061 | 0.0011 | 6.27E-08 |
| bitter alcoholic beverages | rs78119163 | A | G | 0.8838 | 0.0087 | 0.0016 | 7.67E-08 |
| bitter alcoholic beverages | rs28929474 | C | T | 0.9798 | 0.0193 | 0.0036 | 9.24E-08 |
| bitter alcoholic beverages | rs11754010 | T | C | 0.6259 | 0.0056 | 0.001 | 9.86E-08 |
| grapefruit juice | rs139956004 | G | A | 0.0153 | 0.0069 | 0.0014 | 5.57E-07 |
| grapefruit juice | rs117940079 | G | A | 0.0173 | 0.0063 | 0.0013 | 8.00E-07 |
| grapefruit juice | rs62396716 | C | T | 0.8788 | 0.0025 | 5.00E-04 | 9.35E-07 |
| grapefruit juice | rs9986860 | C | T | 0.1303 | 0.0022 | 5.00E-04 | 8.50E-06 |
| grapefruit juice | rs145016664 | T | C | 0.0455 | 0.0035 | 8.00E-04 | 1.51E-05 |
| grapefruit juice | rs146182524 | C | T | 0.0375 | 0.0037 | 9.00E-04 | 2.07E-05 |
| grapefruit juice | rs72881671 | A | G | 0.9306 | 0.0027 | 6.00E-04 | 3.44E-05 |
| grapefruit juice | rs6797526 | T | C | 0.4669 | 0.0014 | 3.00E-04 | 4.07E-05 |

EA, effect allele; EAF, effect allele frequency; NEA, non-effect allele; SNPs, single nucleotide polymorphism.

Supplementary Figure 1. Scatter plots of the 3MR modelsfor smoking per day that are causally related to osteoporosis.


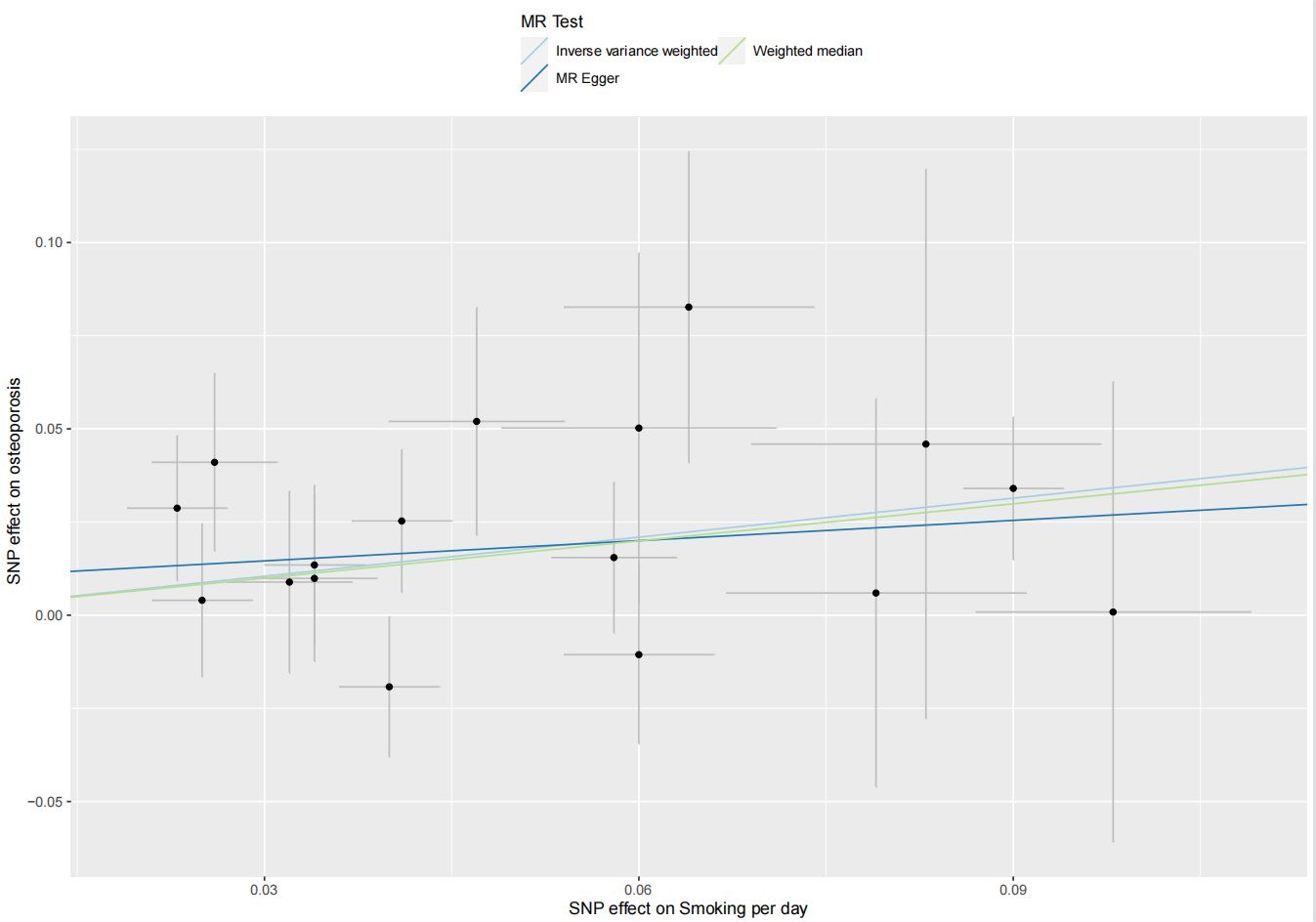


Supplementary Figure 2. Scatter plots of the 3MR modelsfor bitter non-alcoholic beverages that are causally related to osteoporosis.


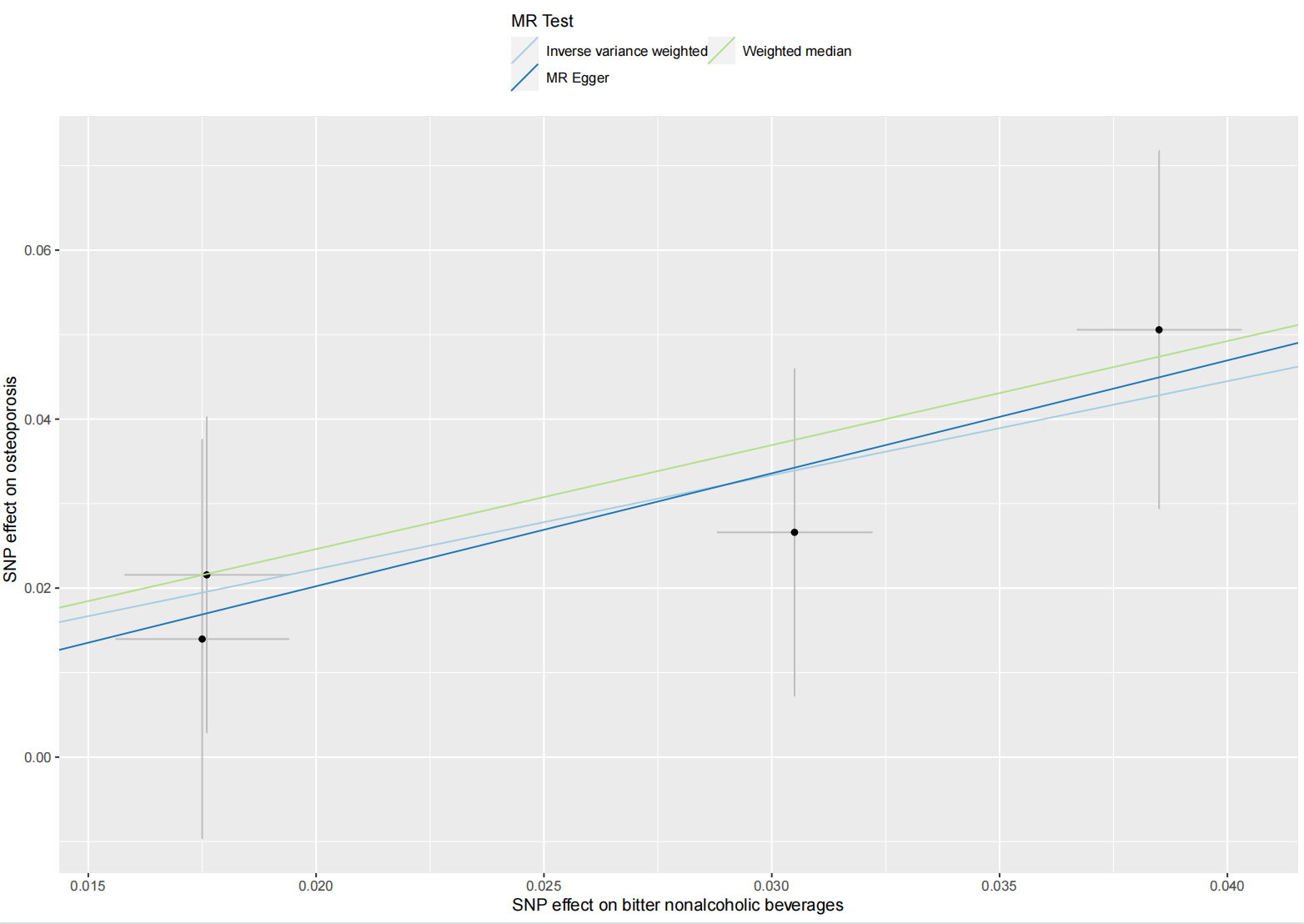


Supplementary Figure 3. Scatter plots of the 3MR modelsfor total bitter beverages that are causally related to osteoporosis.


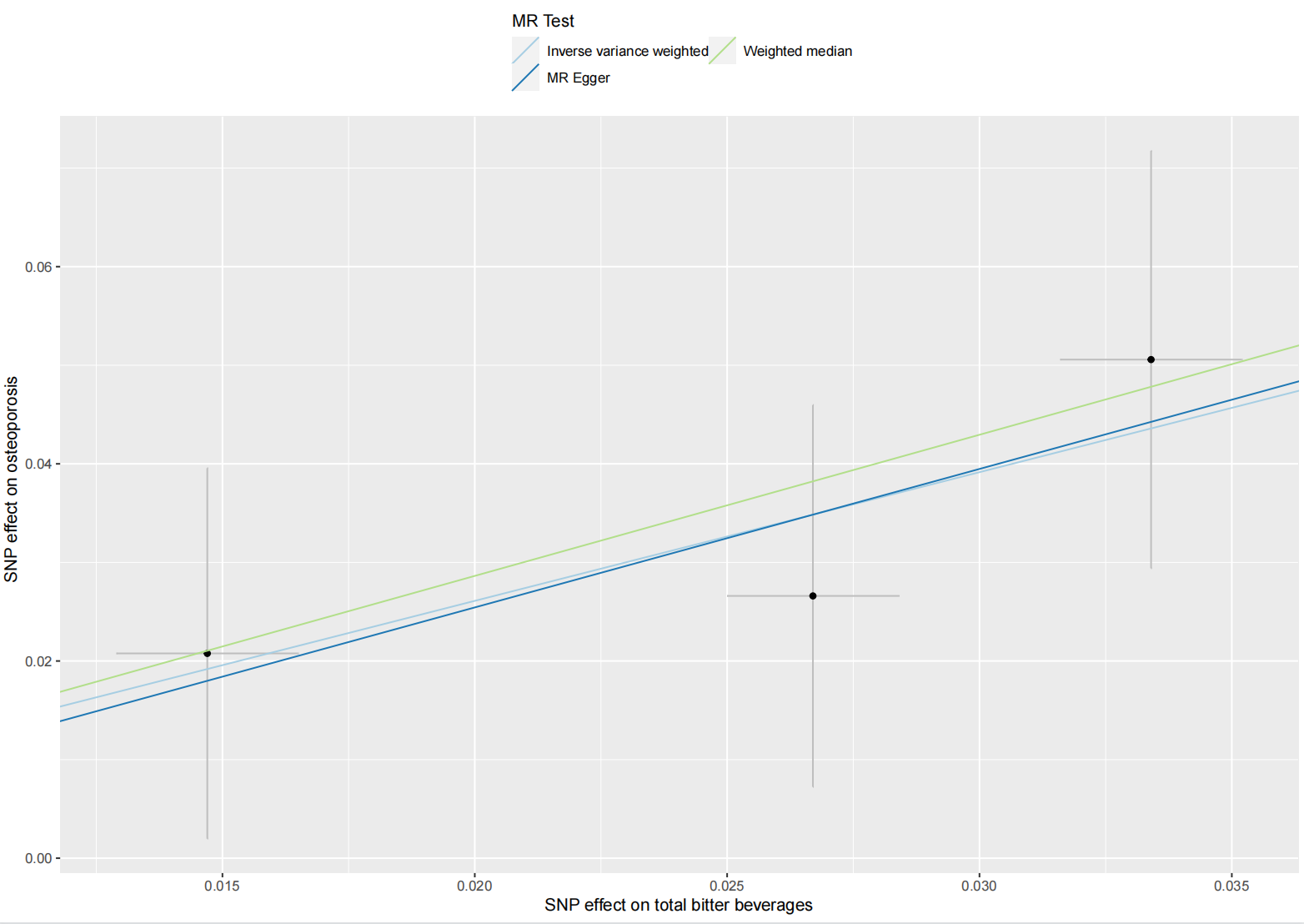


Supplementary Figure 4. Scatter plots of the 3MR modelsfor lifetime smoking index that are causally related to osteoporosis with pathological fracture.


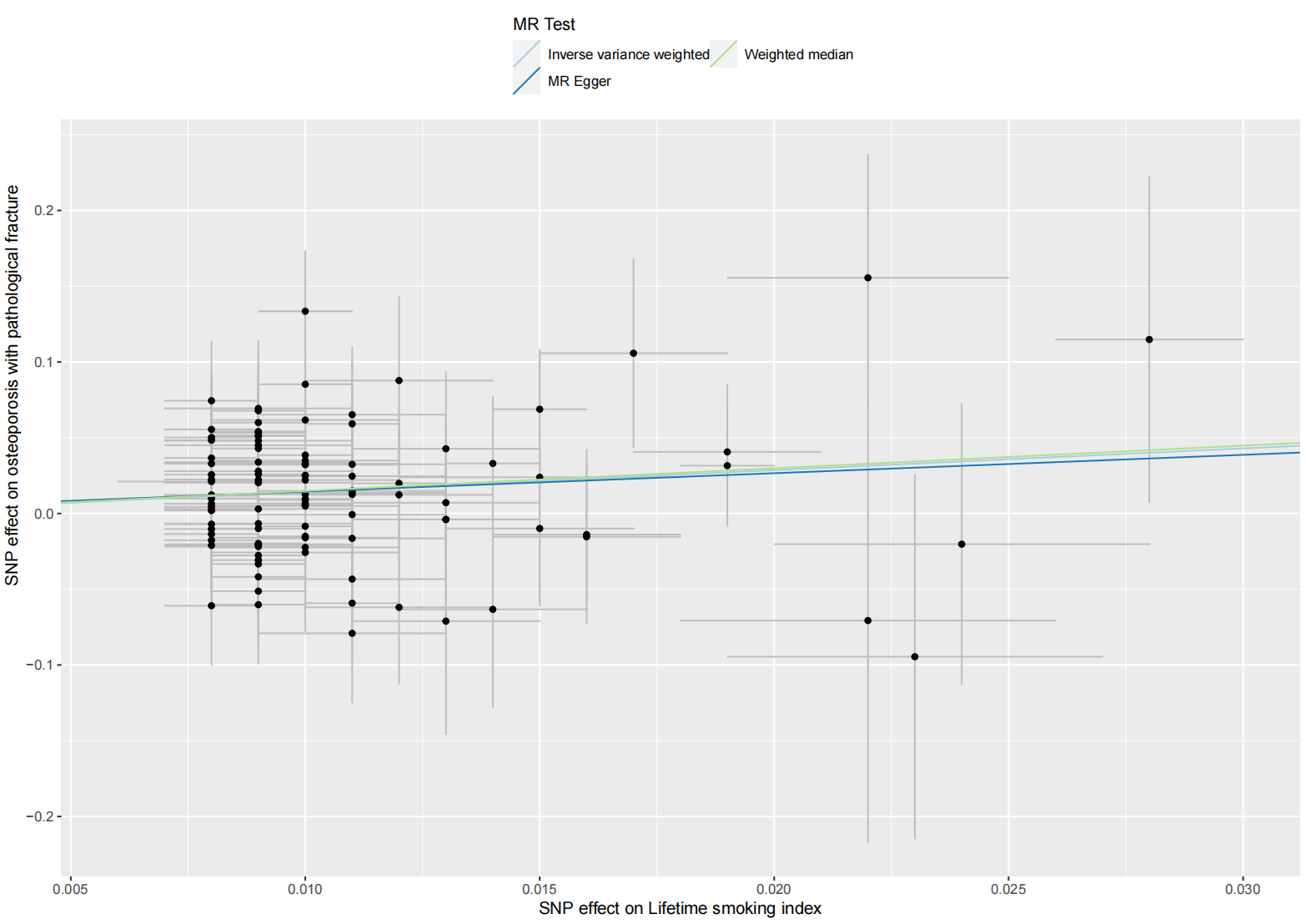


Supplementary Figure 5. The leave-one-out analysis of the causal association between smoking per day and the risk of osteoporosis.


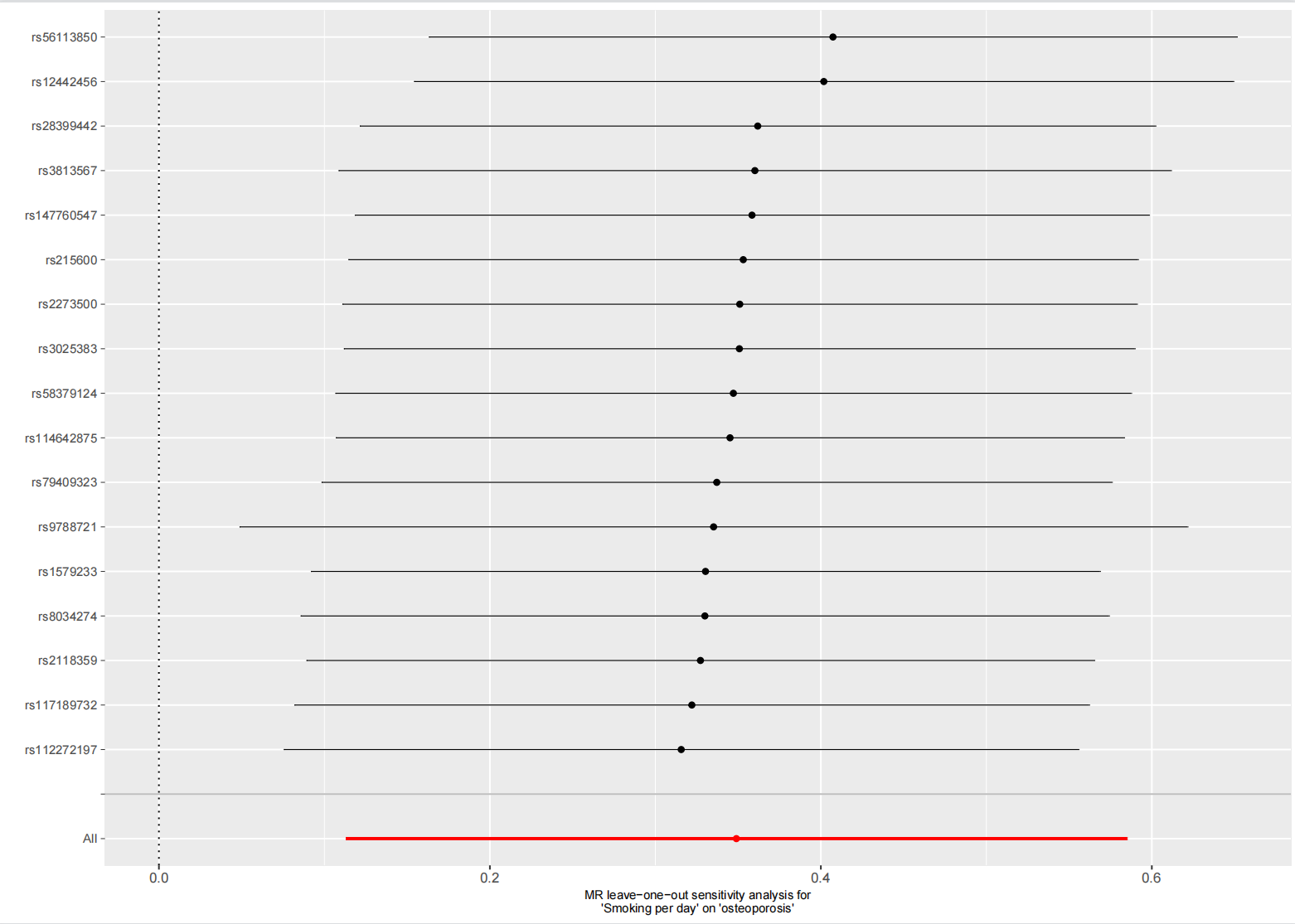


Supplementary Figure 6. The leave-one-out analysis of the causal association between bitter non-alcoholic beverages consumption and the risk of osteoporosis.


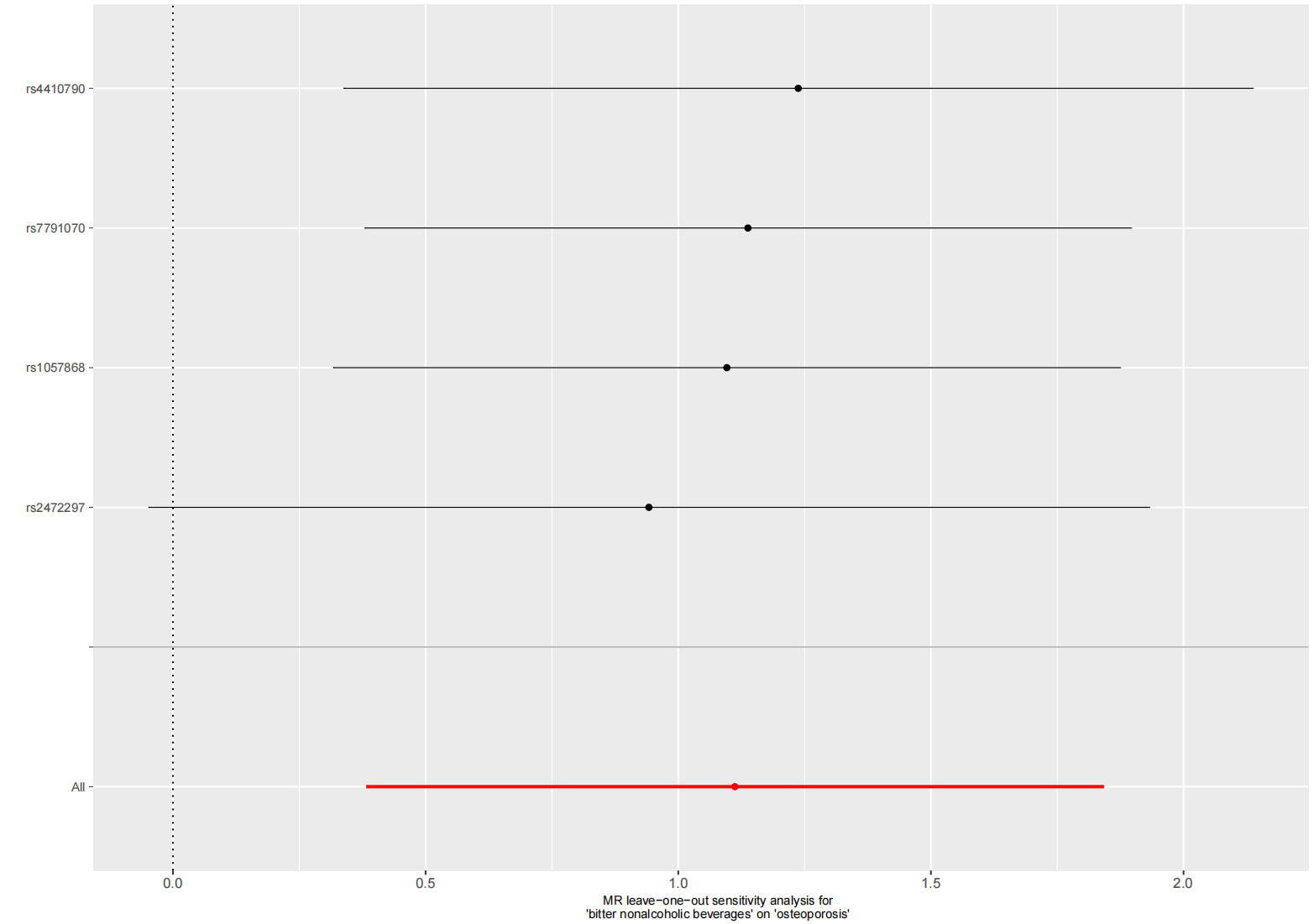


Supplementary Figure 7. The leave-one-out analysis of the causal association between total bitter beverages consumption and the risk of osteoporosis.


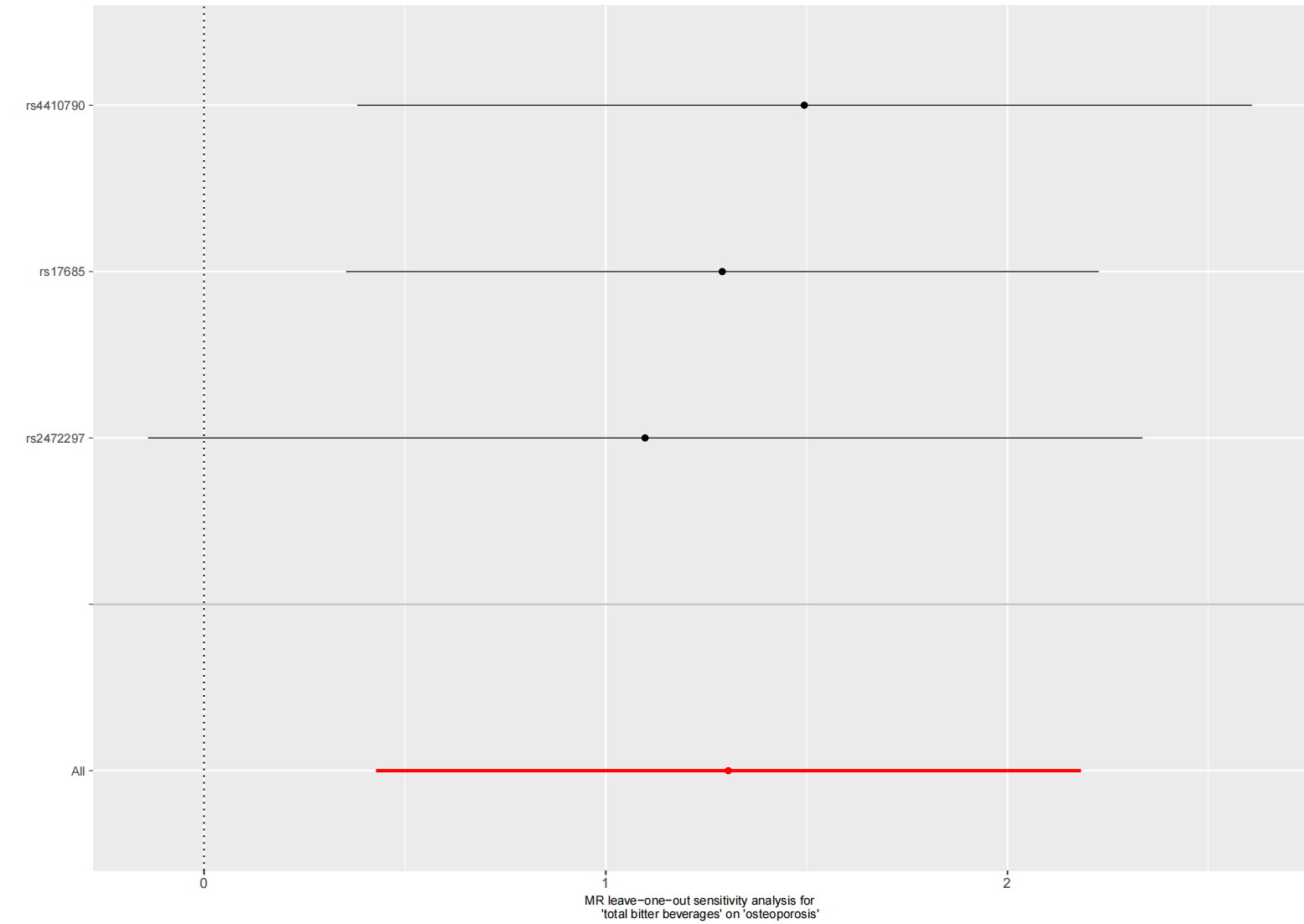


Supplementary Figure 8. The leave-one-out analysis of the causal association between lifetime smoking index and the risk of osteoporosis with pathological fractures.


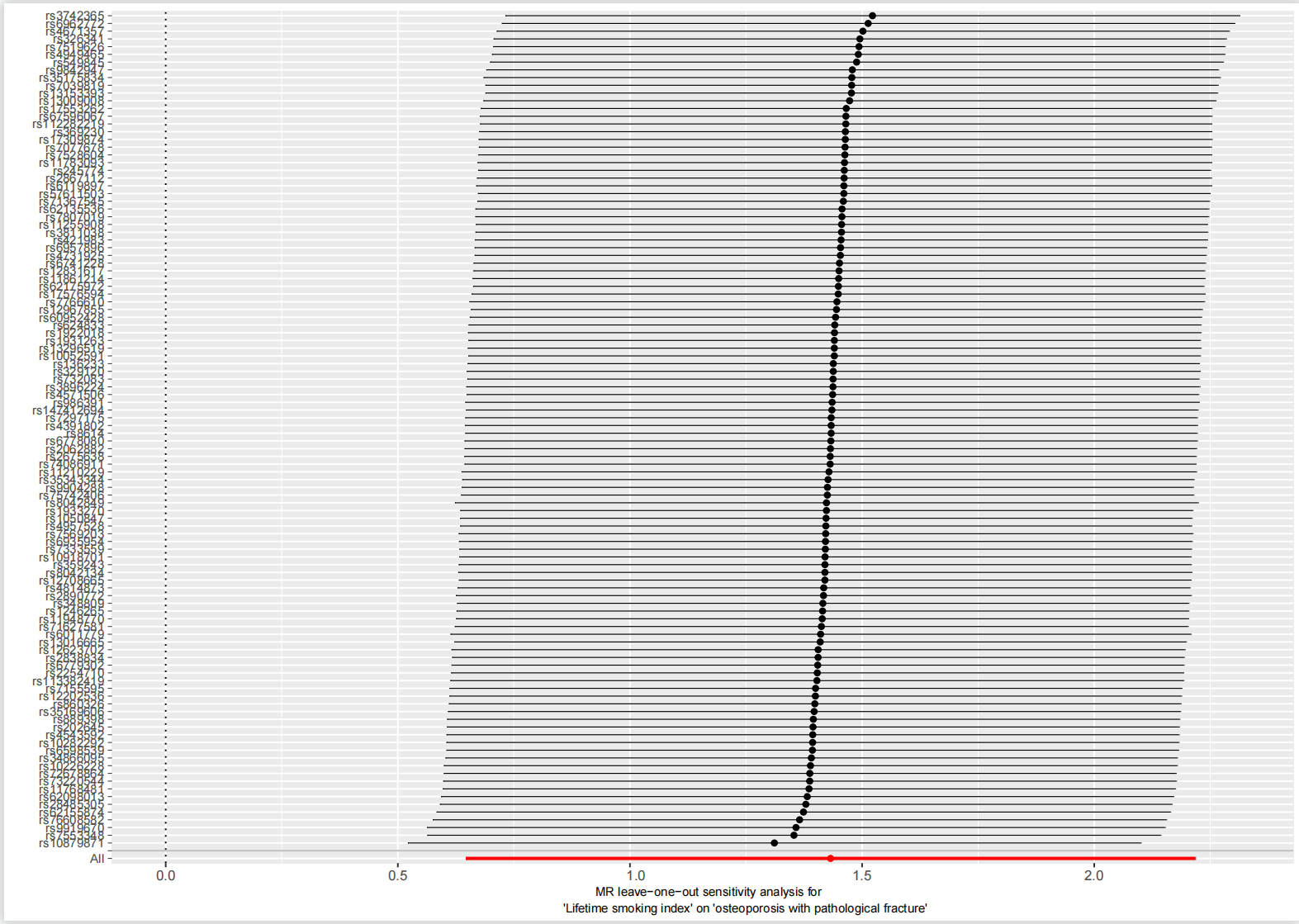

Supplement: Supplementary file 1 — Supplementary Material 1: Supplementary Table 1. STROBE-MR checklist of recommended items to address in reports of Mendelian randomization studies. Supplementary Table 2. Detailed information on used studies. Supplementary Table 3. Detailed information on genetic instruments. Supplementary Fig. 1. Scatter plots of the 3MR modelsfor smoking per day that are causally related to osteoporosis. Supplementary Fig. 2. Scatter plots of the 3MR modelsfor bitter non-alcoholic beverages that are causally related to osteoporosis. Supplementary Fig. 3. Scatter plots of the 3MR modelsfor total bitter beverages that are causally related to osteoporosis. Supplementary Fig. 4. Scatter plots of the 3MR modelsfor lifetime smoking index that are causally related to osteoporosis with pathological fracture. Supplementary Fig. 5. The leave-one-out analysis of the causal association between smoking per day and the risk of osteoporosis. Supplementary Fig. 6. The leave-one-out analysis of the causal association between bitter non-alcoholic beverages consumption and the risk of osteoporosis. Supplementary Fig. 7. The leave-one-out analysis of the causal association between total bitter beverages consumption and the risk of osteoporosis. Supplementary Fig. 8. The leave-one-out analysis of the causal association between lifetime smoking index and the risk of osteoporosis with pathological fractures. [file 41065_2025_371_MOESM1_ESM.doc]
